# Effects and safety of *Ophiocordyceps sinensis* preparation in the adjuvant treatment for dialysis patients: a systematic review and meta-analysis

**DOI:** 10.3389/fphar.2024.1360997

**Published:** 2024-07-19

**Authors:** Meixi Liu, Chengji Cui, Tianying Chang, Qingshan Zhou, Yingzi Cui, Shoulin Zhang, Xing Liao

**Affiliations:** ^1^ College of Chinese Medicine, Changchun University of Chinese Medicine, Changchun, China; ^2^ Nephropathy Department, The Affiliated Hospital to Changchun University of Chinese Medicine, Changchun, China; ^3^ Evidence-Based Medicine Office, The Affiliated Hospital to Changchun University of Chinese Medicine, Changchun, China; ^4^ Institute of Clinical Basic Medicine of Chinese Medicine, China Academy of Chinese Medical Sciences, Beijing, China

**Keywords:** dialysis, end-stage renal disease (ESRD), meta-analysis, *Ophiocordyceps sinensis* preparation, randomized controlled trials, systematic review

## Abstract

**Ethnopharmacological relevance:**

*Ophiocordyceps sinensis* (*O. sinensis*), a genus of ascomycete fungi, has been widedly used in China as a dietary supplement or natural remedy and intensively studied in various disease models with its immunomodulatory potentials. It is a rich source of various bioactive compounds and used for treating end-stage renal disease. This systematic review with clinical evidence aimed to highlight the efficacy and safety of *O. Sinensis* as an adjuvant treatment for patients undergoing dialysis.

**Materials and methods:**

A systematic search through nine electronic databases up to 31 April 2024, was conducted for related studies. The Cochrane risk-of-bias tool was used to evaluate the quality of studies. The Grading of Recommendations Assessment, Development, and Evaluation system was used to assess the certainty of evidence. Two researchers independently searched the literature and evaluated the risk of bias.

**Results:**

After the screening, 35 randomized controlled trials (RCTs) involving 2,914 patients were eventually included. The meta-analysis showed that using *O. sinensis* effectively reduced the following outcomes in patients undergoing dialysis: C-reactive protein (15RCTs, MD = −2.22, 95% CI −3.24 to −1.20; very low certainty evidence); creatinine (22RCTs, MD =1.33, 95% CI −1.79 to −0.87; very low certainty evidence); blood urea nitrogen (21RCTs, MD = −1.57, 95% CI −2.07 to −1.07; low certainty evidence);. It could also effectively improve the following outcomes in patients undergoing dialysis: albumin (20RCTs, MD = −0.81, 95% CI −1.21 to −0.41; low certainty evidence); hemoglobin (19RCTs, MD = −1.00, 95% CI −1.43 to −0.57; low certainty evidence). The rate of adverse drug reactions was higher in the control group than in the experimental group (4RCTs, MD = 1.81, 95% CI 0.88–3.74).

**Conclusion:**

The current evidence indicates that patients with dialysis receiving *O. sinensis* in the adjuvant treatment may improve nutritional and micro-inflammatory status and renal function for both hemodialysis and peritoneal dialysis patients. However, some limitation affected the generalizability of our findings. High-quality studies evaluating mortality outcomes of patients with different dialytic modalities in CKD are warranted in future.

**Systematic Review Registration:**

https://www.crd.york.ac.uk/prospero/display_record.php?ID=CRD42022324508, registration number CRD42022324508.

## 1 Introduction

Dialysis is a treatment that removes wastes and extra fluid from the patient’s blood when the patient’s kidneys are no longer able to work effectively (Hakim and Lazarus, 1995). Patients need dialysis when they develop end-stage kidney failure. Usually, by that time, they lose about 85%–90% of their kidney function and have a glomerular filtration rate that falls below 15 mL/(min · 1.73 m^2^) (Tattersall et al., 2011). Dialysis has two types: hemodialysis (HD) using a machine/artificial kidney-like apparatus and peritoneal dialysis using a peritoneal membrane as a filter. HD is done for patients with no residual renal function, whereas peritoneal dialysis (PD) is recommended for younger patients due to its flexibility. In chronic or end-stage kidney failure, dialysis is the best method to remove accumulated toxins from the body and improve the quality of life for the rest of life. However, individuals suffering from CRF, who are on dialysis, may have increased cardiovascular and metabolic risk and an increased risk of getting an infection (Vadakedath and Kandi, 2017). Dialysis vintage is associated with an enhanced risk of death, with each additional year of dialysis treatment associated with an increase in the risk of dying by approximately 6% (Chertow et al., 2000). Based on the United States Renal Data System (USRDS) report, the adjusted survival rate for patients receiving HD is 57% 3 years after the onset of ESKD compared with 68% for patients receiving PD. The 5-year survival for patients receiving HD and PD is 42% and 52%, respectively (System, 2018).

Among patients with maintenance dialysis, the mortality rate is high at about 165/1,000 (Saran et al., 2020). Many patients develop malnutrition and a micro-inflammatory state due to tubing during dialysis, reduced food intake and intestinal digestion and absorption, and metabolic acidosis (Kiebalo et al., 2020; Sahathevan et al., 2020). Numerous complications also affect patients’ quality of life and increase mortality. Therefore, improving the complications is extremely important for prolonging the lifespan of patients and improving their quality of life.

*Ophiocordyceps sinensis* (*O. sinensis*), also named Chinese caterpillar fungus, is a precious traditional medicine mainly distributed on the Qinghai–Tibetan Plateau (Wei et al., 2021). It has become one of the most valuable biological commodities widely traded in recent years worldwide owing to its medicinal values in terms of anti-fatigue, antitumor, and kidney protection (Liu et al., 2019a; Lee et al., 2021; Long et al., 2021). Modern pharmacological experiments found that the main components of *O. sinensis* included cordyceps polysaccharide, cordycepin, cordycepic acid, and so forth (He et al., 2020; Su et al., 2020). *O. sinensis* preparations is overexploited due to the increase in vulnerability and risk for the wild *O. sinensis* (overexploitation and habitat loss) (Wei et al., 2021) and its surged price (Zhang et al., 2020a), which leads to artificial cultivation to make O. sinensis a more affordable material for commercial trade (Yue et al., 2013a). Synthetic *O. sinensis* preparation is made from strains extracted from *O. sinensis* (Liu et al., 2019b). Studies shown that synthetic *O. sinensis* preparations can benefit patients undergoing dialysis by improving their quality of life, reducing the incidence of cardiovascular events, improving the micro-inflammatory state and malnutrition, and so forth (Liu, 2012; Ashraf et al., 2020; Li et al., 2020). Although a systematic review was published in 2019 to evaluate the efficacy of *Cordyceps sinensis* as an adjunctive treatment in patients undergoing HD, (Bee and Zoriah, 2019), we aimed to conduct a comprehensive and updated systematic review and meta-analysis to evaluate the efficacy and safety of *O. sinensis* preparation in both patients undergoing HD and those undergoing PD.

## 2 Objectives

This systematic review aimed to clarify whether *O. sinensis* preparation in the adjuvant treatment for both patients undergoing HD and those undergoing PD was more effective than the control in anti-infection and reducing cardiovascular events. Our secondary objective was to explore the efficacy of the OS in the two dialysis modalities (HD and PD), various sample size, different treatment course, and follow-up period.

## 3 Methods and analysis

### 3.1 Registration

We drafted the protocol according to the Preferred Reporting Items for Systematic Reviews and Meta-Analysis Protocol (PRISMA-P) (Moher et al., 2015). Also, we reported this systematic review in adherence to the Preferred Reporting Items for Systematic Reviews and Meta-Analyses (PRISMA) (Page et al., 2021). The protocol of the curent review has been registered in the International Prospective Register of Systematic Reviews with the identifier CRD42022324508.

### 3.2 Eligibility criteria

The eligibility criteria were formaulated in the Population, Intervention, Comparison, Outcomes, and Study (PICOS) framework as follows.

#### 3.2.1 Type of study

Only randomized controlled trials (RCTs), with or without blinding, that were published in English or Chinese in peer-reviewed journals were included in this review.

#### 3.2.2 Participants

The study included adult participants aged ≥ 18 years who receive HD or PD, regardless of their primary disease, race, gender, and ethnicity.

#### 3.2.3 Intervention

*O. sinensis* preparations were taken orally combined with dialysis and conventional treatments. No restrictions were imposed on the dosage form, administration, course, or manufacturer. Furthermore, 11 kinds of *O. sinensis* preparations were identified which are commonly used to treat patients with dialysis, including Bailing Tablets (capsules), Jinshuibao Tablets (capsules), Zhiling Capsules, *Cordyceps militaris* capsules, *Cordyceps militaris* powder, cultured *C. sinensis* powder, powdered *Cordyceps mortierella* mycelia, *Cordyceps cephalosporium* mycelia, and fermentative *Cordycepis* fungal powder. All of them were approved by the National Medical Products Administration in China.

#### 3.2.4 Comparator

The control group received the same dialysis and conventional treatments as the experimental group. The conventional therapies included low purine, low salt, low fat, low phosphorus quality, a low-protein diet, limited water intake, control of blood pressure, blood lipids, and blood glucose, and the symptomatic treatment for the complications. The study with the control group using other traditional Chinese medicine treatments, including Chinese patent medicine and acupuncture was excluded.

#### 3.2.5 Type of outcomes

After searching the Core Outcome Measures in Effectiveness Trials (COMET, https://www.cometinitia-tive.org/), we used the outcomes from the Standardized Outcomes in Nephrology-Hemodialysis Dialysis (SONG-HD) and Standardized Outcomes in Nephrology-Peritoneal Dialysis (SONG-PD) core outcome sets (Evangelidis et al., 2017; Manera et al., 2020), which were developed by the Standardized Outcomes in Nephrology-Peritoneal Dialysis (Nephrology-Hemodialysis) initiative. Some of the outcomes were selected for the current reviews, which were divided into primary outcomes (e.g., mortality, CVD, and infection) and secondary outcomes (e.g., vascular access problems, dialysis adequacy, hyperkalemia, and life participation). When the included studies did report the aforementioned outcomes, we used alternative outcomes for meta-analysis.

### 3.3 Search strategy

A search strategy was created with the help of an experienced librarian and adapted for searching the databases, including PubMed, Embase, the Cochrane Library, SinoMed, CNKI, VIP, Wanfang Data and International Clinical Trials Register Search Portal, and ClinicalTrials.gov. Finally, we identified RCTs involving the aforementioned interventions. We conducted the literature search from the inception of all the databases to 31 October 2022, and updated the search on 31 April2024. Studies in accordance with the PICOS were considered. Key search terms (MeSH and free words) used for our searches were “Renal Dialysis” or “*O. sinensis*” or “RCTs” or “*Cordyceps*.” The detailed search strategy for all databases is presented in Supplementary Table S1.

### 3.4 Study selection

All retrieved records were imported into the Endnote X9.1 software, and the duplicated records were removed. By referring to the eligibility criteria, two researchers (MXL and TYC) independently (1) screened the titles and abstracts of deduplicated studies and removed those that did not meet the eligibility criteria and (2) then rechecked the full texts of the remaining articles and finally included or excluded. A third reviewer (XL) was consulted in the case of disagreement. All excluded studies during the full-text checking were recorded and tabulated with their justification for exclusion (Supplementary Table S1). The selection process followed the PRISMA flow diagram (Moher et al., 2015).

### 3.5 Data extraction

We extracted information from the included studies, and two researchers (MXL and TYC) filled the extracted data in a pre-designed form designed using an Excel spreadsheet. The data extraction table had information as follows (Tables 2, 3).

1. Study characteristics: published title, author name, journal name, the country where the study was conducted, year of publication, language, sample size, study design, study period, and follow-up period.
2. Participants: male–female ratio, average age, primary disease, disease stage, severity, average duration of disease, and mean history of dialysis.
3. Interventions: hemodialysis or PD; dialysis time, frequency, and duration; comorbidity.
4. Outcomes: Primary outcomes: mortality, CVD, and infection; secondary outcomes: vascular access problems, dialysis adequacy, hyperkalemia, and life participation.

Two researchers (MXL and CJC) independently extracted data from all studies that met the inclusion criteria. All results were cross-examined. When the cross-examination results were inconsistent, the discussion would resolve the disagreement until a consensus was reached or by consulting a third author (TYC and YZC). We contacted the author by phone or email if the critical data of the included study were unavailable or only partly available.

### 3.6 Assessing risk of bias

Two researchers (MXL and TYC) independently assessed the risk of bias for included studies according to the Cochrane risk-of-bias (ROB) tool for interventions (Higgins et al., 2011). ROB consisted of seven domains on which biases within trials were assessed: (1) sequence generation, (2) allocation concealment, (3) blinding of participants and personnel, (4) blinding of outcome assessment, (5) incomplete outcome data, (6) selective reporting, and (7) other biases (baseline imbalance between groups of participants, blocked randomization in trials that were not blinded, and differential diagnostic activity). Each domain was rated as “high,” “unclear,” or “low” risk of bias and reported separately. The assessment was graphed, and Review Manager 5.3 software was used.

### 3.7 Method for data synthesis

Qualitative evidence synthesis was performed based on the available results. After describing the baseline characteristics of the studies, the outcome of interest was summarized, that is, the effects of *O. sinensis* preparations in the adjuvant treatment for patients undergoing HD and those undergoing PD. Furthermore, the effect evaluation for patients undergoing HD and those undergoing PD was performed separately. Statistical significance was set at *p* < 0.05.

#### 3.7.1 Meta-analysis

A meta-analysis was conducted when the number of RCTs corresponded to the same PICOS in two or more. Effect sizes were calculated as either OR (for dichotomous data) and weighted (or standardized) final post-intervention mean differences (for continuous data) with their corresponding 95% confidence intervals. Review Manager 5.3 software (Program, 2014) was used to conduct meta-analyses. The effects models (fixed or random) were used to estimate the effect of *O. sinensis* preparation by creating forest plots. When heterogeneity was present, the random-effects model was used.

#### 3.7.2 Heterogeneity assessment

We estimated the between-study heterogeneity in all eligible comparisons, used the *χ* ^2^-based Q statistic (Cohen et al., 2015), and assessed the extent of heterogeneity with *I* ^2^, a quantitative measure of inconsistency between studies. When the values were 0% or ≥50%, they represented no heterogeneity or considerable heterogeneity, respectively (Higgins and Thompson, 2002). If the heterogeneity was within the acceptable range, the fixed-effects model was used to affect estimates; otherwise, the random-effects model was used.

#### 3.7.3 Publication bias

We assessed publication bias using funnel plots and Egger tests because more than 10 studies were included in the meta-analysis (Guyatt et al., 2011a). If the funnel plot showed asymmetry, it indicated publication bias. If publication bias existed, trim-and-fill analyses were used to assess the impact of publication bias on the results. Any bias was explained through the analyses and discussions.

#### 3.7.4 Sensitivity analysis

The sensitivity analysis was performed to verify the robustness of the results. We performed this through the leave-one-out strategy (Banach et al., 2015a; Banach et al., 2015b) based on the quality of the included studies to explore the sources of heterogeneity. When one study was excluded, the results and heterogeneity of the remaining studies were reevaluated.

#### 3.7.5 Subgroup analysis

The subgroup analysis was conducted to analyze the causes of heterogeneity. We performed this based on the two dialysis modalities (HD and PD), sample size, treatment course, and follow-up period.

### 3.8 Quality of the evidence

The certainty of the evidence was graded for each outcome, from a rating of HIGH to VERY LOW, by following the Grading of Recommendations Assessment, Development, and Evaluation (GRADE) approach (Guyatt et al., 2011b). The GRADE system included five domains that could downgrade the quality of the evidence used in RCTs: limitations, inconsistent results, imprecision, indirectness, and publication bias. The quality of evidence for each outcome was graded as HIGH, MODERATE, LOW, or VERY LOW. A summary of findings (SoF) was created using GRADEPro GDT 2021 (McMaster University, ON, Canada). The SoF presented the following information where appropriate: absolute risks for the treatment and control, estimates of relative risk, and a ranking of the quality of the evidence based on the risk of bias, directness, heterogeneity, precision, and risk of publication bias of the review results. The outcomes reported in the SoF table for this review included CVD, mortality, dialysis adequacy, infection, and so forth.

## 4 Results

### 4.1 Literature search

**FIGURE 1:**
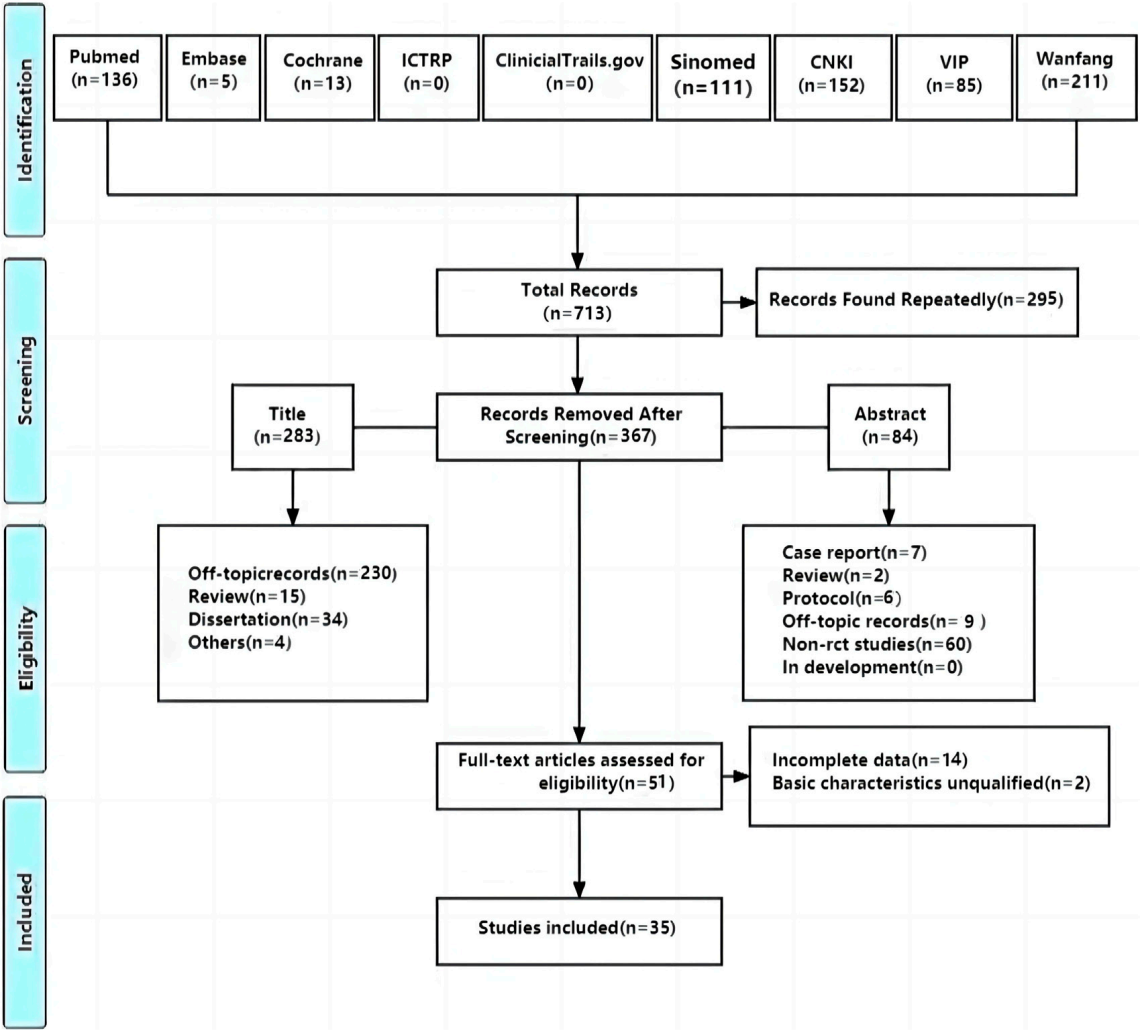
PRISMA flow diagram of the study selecting process.

A total of 713 studies were retrieved, and 35 studies (Sun et al., 2011; Sun et al., 2012; Wu, 2012; Ye et al., 2012; Liu, 2014; Tian et al., 2014; Wang, 2016; Yao et al., 2016; Chen et al., 2017; Huang et al., 2018; Wang and Sha, 2018; Ying, 2018; Gao and Gao, 2019; Wang, 2019; Wang and Wang, 2019; Xie et al., 2019; Yang et al., 2019; Zhu, 2019; Zhang et al., 2020b; Cui et al., 2020; Zhang et al., 2020c; Zhu and Yu, 2020; He, 2021; Li, 2021; Ling et al., 2021; Ren, 2021; Tian et al., 2021; Gao et al., 2022; Zhang et al., 2022; Zhao et al., 2022; Li et al., 2023; Wang et al., 2023; Zeng, 2023; Lin et al., 2024; Tang and Shao, 2024) with 2,914 patients were included. Further, 295 studies were screened out because of duplication, 367 were excluded after reading titles and abstracts, and 51 were assessed for eligibility by reading full texts. After that, 14 RCTs were excluded for incomplete data and 2 were excluded for unqualified basic characteristics. Ultimately, 35 RCTs were included to conduct meta-analysis (Figure 1).

### 4.2 Description of the included studies

**FIGURE 2:**
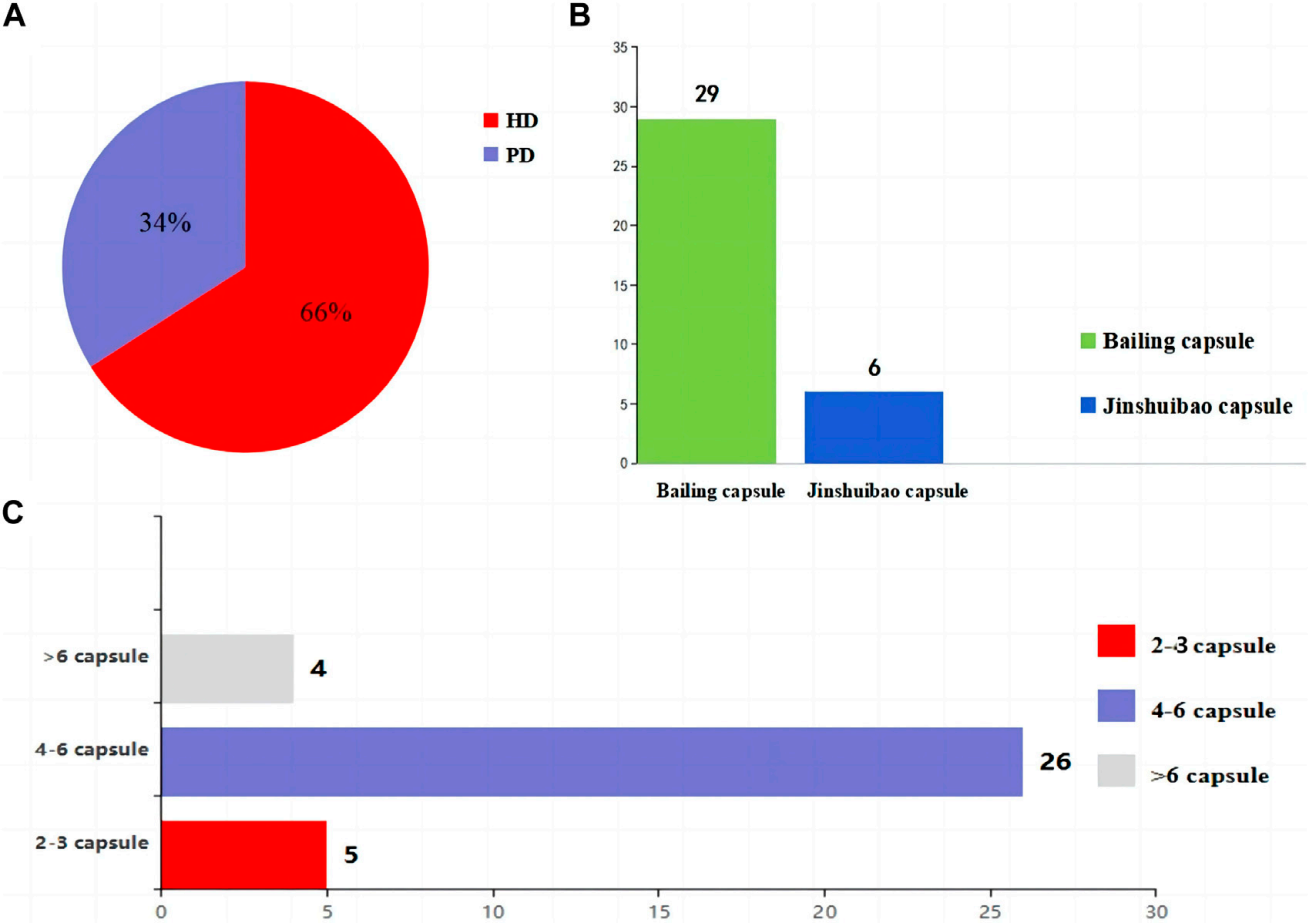
The information about the intervention. **(A)** Selection of the dialysis method in the patients. **(B)** *O. sinensis* preparations. **(C)** The doses of *O. sinensis* preparations. HD, Hemodialysis; PD, peritoneal dialysis.

The included studies were all from China. The sample size of the included studies ranged from 35 to 150, age from 42.2 ± 15.2 to 74.54 ± 2.06 years, disease duration from 2.21 ± 0.58 to 168.24 ± 21.72 months, and history of dialysis from 10.4 ± 2.0 to 35.16 ± 6.73 months. As for the dialysis modality, 1931 (66%) patients received HD while 983 (34%) patients were treated by PD. In terms of the selection of *O. sinensis* preparations, 29 of 35 studies used the Bailing capsule and 6 studies used the Jinshuibao capsule. For the doses of *O. sinensis* preparations, patients in 5 studies took 2 to 3 capsules at a time, patients in 26 studies took 4 to 6 capsules, and patients in 4 studies took more than 6 capsules (Figure 2; Tables 1, 2).

**TABLE 1:** Basic characteristics of the included studies (Part Ⅰ).

Study	Year of publication	Country	Language	M/W	Average age	Study period (week)	Follow-up period	Drug administration
Li (2021)	2021	China	Chinese	14/11 16/9	66.21 ± 5.05 66.08 ± 5.02	24	ND	5 capsules tid
Gao and Gao (2019)	2019	China	Chinese	26/15 25/16	69.73 ± 3.24 69.34 ± 3.51	12	ND	6 capsules tid
Zhu and Yu (2020)	2020	China	Chinese	22/17 20/19	47.85 ± 3.61 47.81 ± 3.63	24	ND	4–6 capsules tid
Zhu (2019)	2019	China	Chinese	29/16 27/18	51.43 ± 8.27 51.69 ± 8.46	12	ND	4 capsules tid
Tian et al. (2014)	2014	China	Chinese	NS	54.43 ± 11.7	4	ND	2 capsules tid
Tian et al. (2021)	2021	China	Chinese	17/13 16/14	54.6 ± 10.6 53.5 ± 11.5	8	ND	4 capsules tid
Zhang et al. (2020a)	2020	China	Chinese	31/12 28/15	62.7 ± 9.5 63.5 ± 8.3	12	ND	5 capsules tid
Ren (2021)	2021	China	Chinese	29/26 29/26	60.81 ± 3.86 60.98 ± 3.84	8	ND	4 capsules tid
Yang et al. (2019)	2019	China	Chinese	20/16 17/19	54.28 ± 3.89 55.26 ± 3.48	24	ND	4 capsules tid
Huang et al. (2018)	2018	China	Chinese	NS	45.6 ± 12.4	12	ND	2 capsules tid
Cui et al. (2020)	2020	China	Chinese	18/15 20/13 19/15	52.06 ± 8.11 53.15 ± 7.28 53.36 ± 8.20	8	1 year	4 capsules tid
Ling et al. (2021)	2021	China	Chinese	23/18 24/17	59.05 ± 3.29 58.96 ± 3.27	12	ND	4 pills tid
Xie et al. (2019)	2019	China	Chinese	27/23 29/21	NS	24	ND	10 capsules tid
Wang and Sha (2018)	2018	China	Chinese	24/16 23/17	43.16 ± 11.56 46.33 ± 9.14	24	ND	5 capsules tid
Yao et al. (2016)	2016	China	Chinese	11/6 11/7	62.82 ± 8.75 61.61 ± 10.80	24	ND	5 capsules tid
Chen et al. (2017)	2017	China	Chinese	17/8 15/10	47.1 ± 12.3 45.5 ± 10.1	12	ND	6 capsules tid
Sun et al. (2011)	2011	China	Chinese	41/28 23/10	42.2 ± 15.2 43.2 ± 16.2	24	ND	2 capsules tid
Zhang et al. (2020b)	2020	China	Chinese	25/22 27/20	57.86 ± 8.29 59.13 ± 6.87	12	ND	5 capsules tid
He (2021)	2021	China	Chinese	21/17 22/16	52.19 ± 7.59 52.65 ± 7.37	12	ND	4 capsules tid
Sun et al. (2012)	2012	China	Chinese	31/22 33/16	52 ± 14 57 ± 9	24	ND	2 capsules tid
Ying (2018)	2018	China	Chinese	37/10 36/11	57.83 ± 5.46 58.71 ± 5.72	24	ND	4 capsules tid
Wang (2016)	2016	China	Chinese	17/13 16/14	53.4 ± 8.3 52.6 ± 8.7	24	ND	10 capsules tid
Ye et al. (2012)	2012	China	Chinese	19/15 18/16	61.6 ± 9.3 61.3 ± 8.8	12	ND	6 capsules tid
Liu (2014)	2014	China	Chinese	16/12 15/13	54.6 ± 7.8 53.7 ± 8.2	24	ND	10 capsules tid
Wang (2019)	2019	China	Chinese	40/35 38/37	52.6 ± 13.5 53.9 ± 14.7	4	ND	10 capsules tid
Gao et al. (2022)	2022	China	Chinese	ND	ND	4	1 year	5 capsules tid
Wang and Wang (2019)	2019	China	Chinese	17/11 19/9	55.2 ± 3.5 53.9 ± 4.7	24	ND	4 capsules tid
Wu (2012)	2012	China	Chinese	14/7 15/6 15/6	45.10 ± 5.51 43.58 ± 6.32 44.18 ± 5.09	8	ND	4 capsules tid
Lin et al. (2024)	2024	China	Chinese	25/15 24/16	51.38 ± 2.56 50.42 ± 2.45	12	ND	3 capsules tid
Li et al. (2023)	2023	China	Chinese	48/17 41/24	52.54 ± 4.83 53.48 ± 7.30	12	ND	6 capsules tid
Zeng (2023)	2023	China	Chinese	26/28 27/27	52.51 ± 9.67 53.74 ± 10.81	8	ND	4 capsules tid
Wang et al. (2023)	2023	China	Chinese	24/6 21/9	59.10 ± 8.45 56.92 ± 8.61	16	ND	4 capsules tid
Zhang et al. (2022)	2022	China	Chinese	25/19 27/15	74.54 ± 2.06 74.13 ± 2.19	12	ND	5 capsules tid
Tang and Shao (2024)	2024	China	Chinese	34/29 32/31	57.39 ± 3.15 57.48 ± 3.26	8	ND	6 capsules tid
Zhao et al. (2022)	2022	China	Chinese	26/14 27/13	49.01 ± 4.01 49.03 ± 4.02	12	ND	4 capsules tid

Tid, Three times a day; ND, no data.

**TABLE 2:** Basic clinical characteristics of the included studies (Part Ⅱ).

Study	Therapy method	Sample-size	Average duration of disease (month)	Mean history of dialysis (month)	Comorbidity	Dialysis time	Dialysis frequency (times/week)	Mortality	CVD	Infection	Vascular access problems	Dialysis adequacy	Hyperk-alaemia	Life participa-tion
Li (2021)	E: PD + CT + Bailing capsule C: PD + CT	25 25	83.04 ± 24.48 84.12 ± 24.36	24.12 ± 7.44 23.76 ± 7.20	ND	ND	ND	ND	ND	ND	ND	ND	ND	QOL-BREFL scale
Gao and Gao (2019)	E: HD + CT + Bailing capsule C: HD + CT	41 41	ND	34.52 ± 5.46 35.16 ± 6.73	ND	4 h	3	ND	ND	ND	ND	ND	ND	ND
Zhu and Yu (2020)	E: PD + Bailing capsule+ Levocarnitine C: PD + levocarnitine	39 39	ND	16.85 ± 2.10 16.89 ± 2.07	E: CPN:2 PKD: 5 D N:12 CGN: 20 C: CPN: 3 PKD: 5 D N:13 CGN: 18	ND	3–4	ND	ND	ND	ND	ND	ND	ND
Zhu (2019)	E: HD + CT + Bailing capsule C: HD + CT	45 45	68.28 ± 15.24 66.36 ± 16.08	ND	E: HRD: 10 DN: 12 CGN:17 PKD: 6 C: HRD: 9 DN: 11 CGN: 20 PKD: 5	ND	ND	ND	ND	ND	ND	ND	ND	ND
Tian et al. (2014)	E: PD + CT + Bailing capsule C: PD + CT	30 28	ND	ND	DN	ND	7	ND	ND	ND	ND	ND	ND	ND
Tian et al. (2021)	E: PD + CT + Bailing capsule C: PD + CT	30 30	98.40 ± 26.04 103.08 ± 26.76	ND	ND	ND	7	ND	ND	ND	ND	ND	ND	ND
Zhang et al. (2020a)	E: PD + CT + Bailing capsule C: PD + CT	43 43	68.40 ± 10.80 66.00 ± 9.60	10.40 ± 2.00 10.70 ± 2.10	DN	ND	7	ND	ND	ND	ND	ND	ND	ND
Ren (2021)	E: HD + CT + Bailing capsule C: HD + CT	55 55	10.56 ± 2.16 10.80 ± 2.16	ND	ND	ND	3–4	ND	ND	ND	ND	ND	ND	ND
Yang et al. (2019)	E: HD + CT + Bailing capsule + Levocarnitine C: HD + CT	36 36	58.56 ± 19.80 63.12 ± 17.64	19.48 ± 3.01 20.18 ± 2.69	E: CGN:9 DN:7 HRD:5 PKD:7 IRD:5 ON:3 C: CGN:8 DN:9 HRD:7 PKD:5 IRD: 6 ON:1	4 h	3	ND	ND	ND	ND	ND	ND	ND
Huang et al. (2018)	E: HD + CT + Bailing capsule C: HD + CT	50 50	ND	ND	ND	4 h	2–3	ND	ND	ND	ND	ND	ND	ND
Cui et al. (2020)	E: HFHD + Bailing capsule C1: LFHD C2: HFHD	34 33 33	167.64 ± 24.6 168.24 ± 21.72 153.72 ± 18.60	20.02 ± 3.18 18.30 ± 2.11 19.44 ± 2.52	DN	4 h	3	ND	E: 6 (17.65%) C1:19 (57.58%) C2:13 (39.39%)	ND	ND	ND	ND	ND
Ling et al. (2021)	E: HD + Jinshuibao capsule C: HD + Diazepam	41 41	ND	30.18 ± 4.21 30.14 ± 4.19	E: CGN: 17 HRD: 15 ON: 7 DN: 2 C: CGN: 18 HRD: 14 ON: 6 DN: 3	4 h	3	ND	ND	ND	ND	ND	ND	ND
Xie et al. (2019)	E: PD + Bailing capsule + Levocarnitine C: PD + Levocarnitine	50 50	ND	ND	ND	ND	7	ND	ND	ND	ND	ND	ND	ND
Wang and Sha (2018)	E: PD + CT + Bailing capsule C: PD + CT	40 40	40.32 ± 9.24 42.12 ± 11.40	ND	ND	ND	7	ND	ND	ND	ND	E: Before: 0.88 ± 0.26 After: 1.02 ± 0.20 C: Before: 1.15 ± 0.12 After: 1.31 ± 0.20	ND	ND
Yao et al. (2016)	E: PD + Bailing capsule Control: PD	17 18	ND	ND	ND	24 h	7	ND	ND	ND	ND	E (1 month): 1.77 ± 0.10 C (1 month): 1.78 ± 0.08	ND	ND
Chen et al. (2017)	E: HD + CT + Bailing capsule C: HD + CT	25 25	24.00–120.00 24.00–108.00	ND	ND	4 h	4	ND	ND	ND	ND	ND	ND	ND
Sun et al. (2011)	E: HD + CT + Bailing capsule C: HD + CT	69 33	ND	ND	E: CGN: 42 DN: 18 PKD: 3 AASV:2 HRD: 3 LN: 1 C: CGN: 20 DN: 11 PKD: 1 LN: 1	4 h	2–3	ND	ND	ND	ND	ND	ND	ND
Zhang et al. (2020b)	E: PD + CT + Bailing capsule + Compoundα-ketoacid tablets C: PD + CT + Compoundα- ketoacid tablets	47 47	ND	21.43 ± 4.39 22.17 ± 6.54	E: CGN: 29 DN: 12 BANS: 6 C: CGN: 28 DN: 14 BANS: 5	24 h	7	ND	ND	ND	ND	ND	ND	ND
He (2021)	E: HD + EPO + Bailing capsule C: HD + EPO	38 38	ND	24.45 ± 9.75 24.36 ± 10.08	NS	4 h	3	ND	ND	ND	ND	ND	ND	ND
Sun et al. (2012)	E: PD + CT + Bailing capsule + Compound α-ketoacid tablets C: PD + CT	53 49	ND	ND	E: CGN: 38 DN: 16 PKD: 3 AASV: 2 HRD: 3 LN: 1 C: CGN: 30 DN: 15 PKD: 3 LN: 1	ND	3–4	ND	ND	ND	ND	ND	ND	ND
Ying (2018)	E: PD + CT + Bailing capsule + Levocarnitine C: PD + CT + Levocarnitine	47 47	ND	ND	E: CGN: 23 DN: 14 Others: 10 C: CGN: 21 DN: 15 Others: 11	ND	7	ND	ND	ND	ND	ND	ND	ND
Wang (2016)	E: HD + CT + Bailing capsule + Compound α-ketoacid tablets C: HD + CT + Compound α-ketoacid tablets	30 30	ND	ND	ND	ND	ND	ND	ND	ND	ND	ND	ND	ND
Ye et al. (2012)	E: HD + CT + Jinshuibao capsule C: HD + CT	34 34	ND	17.60 ± 10.20 16.90 ± 9.70	DN	4 h	3	ND	ND	ND	ND	ND	ND	ND
Liu (2014)	E: PD + CT + Bailing capsule + Levocarnitine C: PD + CT + Levocarnitine	28 28	ND	ND	E:CGN: 11 PKD: 2 HRD: 3 DN: 7 CPN: 5 C: CGN: 12 PKD: 2 HRD: 2 DN: 6 CPN: 6	ND	ND	ND	ND	ND	ND	ND	ND	ND
Wang (2019)	E: HD + CT + Bailing capsule + Levocarnitine C: HD + CT	75 75	ND	ND	ND	ND	ND	ND	ND	ND	ND	ND	ND	ND
Gao et al. (2022)	E: PD + Bailing capsule C: PD	45 45	ND	ND	ND	ND	ND	ND	ND	ND	ND	E (1 month): 1.58 ± 0.12 C (1 month): 1.69 ± 0.07	ND	ND
Wang and Wang (2019)	E: HD + Bailing capsule C: HD	28 28	ND	ND	E: CGN: 14 DN: 4 BANS: 10 C: CGN: 13 DN: 5 BANS: 10	4 h	3	ND	ND	ND	ND	ND	ND	ND
Wu (2012)	E: LFHD C1: HFHD C2: HFHD + Bailing capsule	21 21 21	ND	ND	ND	4 h	3	ND	ND	ND	ND	ND	ND	ND
Lin et al. (2024)	E: HD + Jinshuibao capsule C: HD	40 40	16.12 ± 3.48 16.37 ± 3.52	ND	E: DN:40 C: DN:40	4 h	3	ND	ND	ND	ND	ND	ND	ND
Li et al. (2023)	E: HD + Bailing capsule C: HD	65 65	62.39 ± 5.19 60.86 ± 6.73	ND	ND	3 h	2–3	ND	ND	ND	ND	ND	ND	ND
Zeng (2023)	E: HD + Bailing capsule + Levocarnitine C: HD + Levocarnitine	54 54	42.24 ± 15.36 42.96 ± 16.08	ND	E: CGN:17 DN: 19 PKD: 4 HRD: 14 C: CGN: 16 DN: 20 PKD: 3 HRD: 15	ND	ND	ND	ND	ND	ND	ND	ND	GQOL-74
Wang et al. (2023)	E:HD + CT + Jinshuibao capsule C: HD + CT	30 30	10.01 ± 1.68 9.23 ± 1.35	ND	E: CGN:6 DN: 5 HRD: 9 C: CGN: 7 DN: 6 HRD: 7	4 h	3	ND	ND	ND	ND	ND	ND	ND
Zhang et al. (2022)	E: HD + Jinshuibao capsule C: HD	44 42	ND	ND	E: DN:44 C: DN:42	4 h	3	ND	ND	ND	ND	ND	ND	ND
Tang and Shao (2024)	E: HD + Jinshuibao capsule C: HD	63 63	ND	ND	E: CGN:21 DN:1 5 HRD: 23 Others:4 C: CGN: 18 DN: 19 HRD: 21 Others:5	4 h	3	ND	ND	ND	ND	ND	ND	ND
Zhao et al. (2022)	E: HD + CT + Bailing capsule C: HD + CT	40 40	26.04 ± 6.0 2.21 ± 0.58	ND	ND	4 h	3	ND	ND	ND	ND	ND	ND	ND

AASV, small-vessel vasculitis; BANS, benign arteriolar nephrosclerosis; CVD, cardiovascular disease.C, control group; CT, conventional treatment; CPN, chronic pyelonephritis; CGN, chronic glomerulonephritis; DN, diabetic nephropathy; E, experimental group; HD, hemodialysis; HRD, hypertensive renal disease; IRD, ischemic renal disease; ND, no data; ON, obstructive nephropathy; PD, peritoneal dialysis; PKD, polycystic kidney.

### 4.3 Risk of bias

**FIGURE 3:**
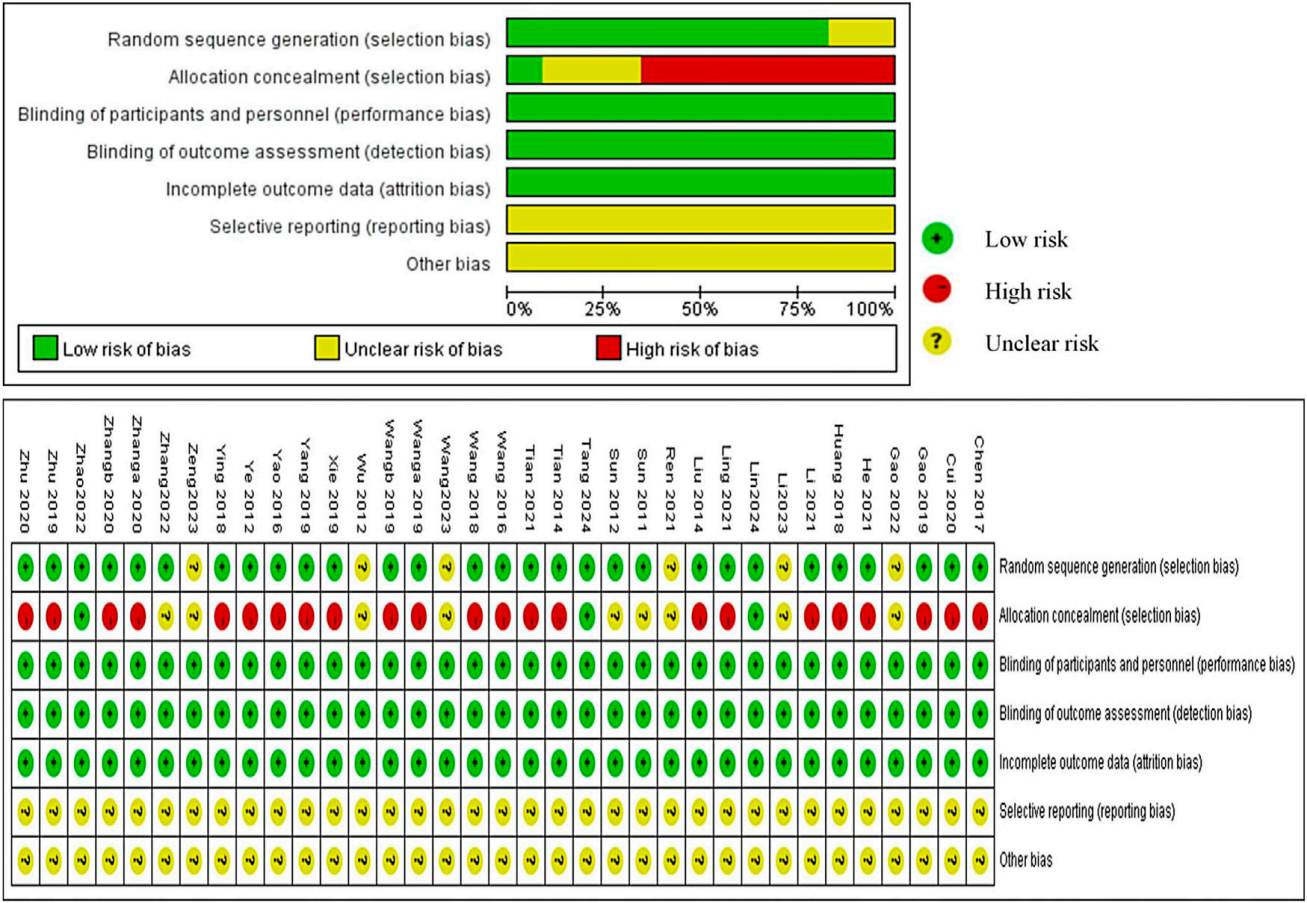
Risk of bias in included studies.

We evaluated 35 RCTs based on the Cochrane ROB tool and found that 30 RCTs used the random sequence generation methods, such as random number table, and 5 studies, due to lack of description, were appraised as” unclear risk” (Wu, 2012; Ren, 2021; Gao et al., 2022; Li et al., 2023; Zeng, 2023). A majority of studies were evaluated as “high risk,” and only nine studies (Sun et al., 2011; Sun et al., 2012; Wu, 2012; Ren, 2021; Gao et al., 2022; Zhang et al., 2022; Li et al., 2023; Wang et al., 2023; Zeng, 2023) were classified as “unclear risk” because of the unclear allocation concealment scheme. Although blinding was not used in any of the studies, the outcome measures were objective and the use of blinding did not affect the evaluation of the results by the system reviewers. Therefore, “Blinding of Participants and Personnel” and “Blinding of Outcome Assessment” were defaulted to “low risk.” All studies had no missing data which were rated as “low risk.” No research proposals were found for any of the studies, and we could not judge whether reporting bias existed due to insufficient information; therefore, it was defined as “unclear.” We were assumed that the “Other bias” were unclear because there was not much time to investigate them for us (Figure 3).

### 4.4 Outcomes in patients undergoing dialysis

#### 4.4.1 CRP

**FIGURE 4:**
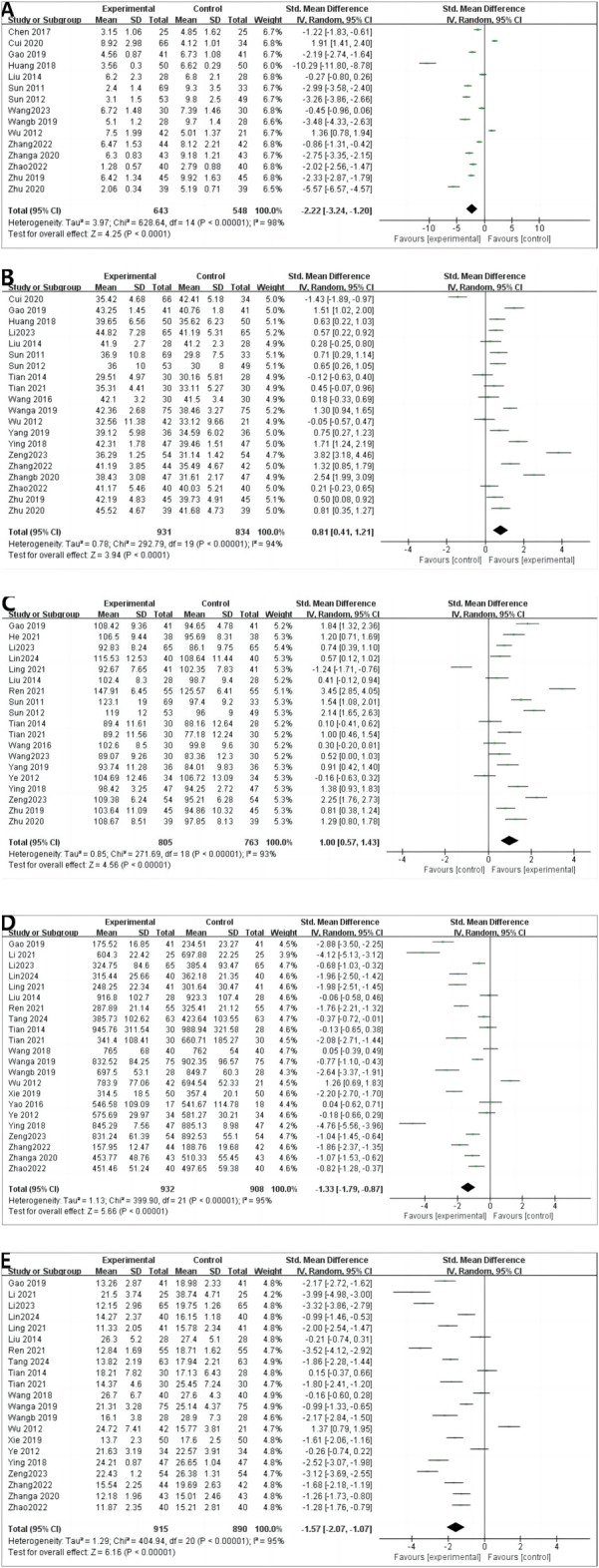
Outcomes in patients undergoing dialysis. **(A)** CRP; **(B)** ALB; **(C)** HGB; **(D)** CREA; **(E)** BUN.

A total of 15 RCTs reported CRP levels before and after treatment; 1,191 patients were included. A random-effects model was used to combine the effect sizes (*p* < 0.00001, *I* ^2^ = 98%). The CRP levels significantly decreased the intervention group compared with in the control group. [MD = −2.22, 95% CI (−3.24 to −1.20), *p* < 0.00001] (Figure 4A).

#### 4.4.2 ALB

A total of 20 RCTs reported ALB levels before and after treatment; 1,765 patients were included. We combined effect sizes using the random-effects model due to large heterogeneity (*p* < 0.00001, *I* ^2^ = 94%). The ALB levels significantly decreased in the intervention group compared with the control group [MD =0.81, 95% CI (0.41–1.21), *p* < 0.0001] (Figure 4B).

#### 4.4.3 HGB

HGB levels were described in 19 RCTs; 1,568 patients were involved. The random-effects model was used to combine effect sizes (*p* < 0.00001, *I* ^2^ = 93%). The HGB levels significantly increased in the intervention group compared with the control group [MD = 1.00, 95% CI (0.57–1.43), *p* < 0.00001] (Figure 4C).

#### 4.4.4 CREA

CREA was reported in 22 RCTs, and 1840 patients were included. The random-effects model was used (*p* < 0.00001, *I* ^2^ = 95%). The CREA levels significantly decreased in the intervention group compared with in control group [MD = −1.33, 95% CI (−1.79 to −0.87), *p* < 0.00001] (Figure 4D).

#### 4.4.5 BUN

A random-effects model was used to conduct a meta-analysis of 21 RCTs that reported BUN involving 1805 patients (*p* < 0.00001, *I* ^2^ = 95%). The BUN levels significantly decreased in the intervention group compared with in [MD = −1.57, 95% CI (−2.07 to −1.07), *p* < 0.00001] (Figure 4E).

#### 4.4.6 Adverse drug reactions

##### 4.4.6.1 Raw incidence of adverse reactions

**FIGURE 5:**
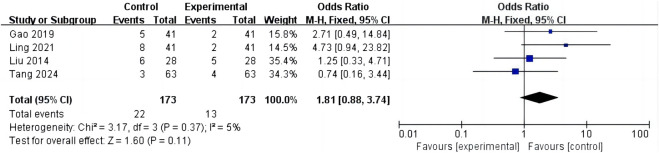
Forest plot of adverse drug reactions.

A total of 4 studies and 346 patients were enrolled. Data were pooled using a fixed-effects model (*p* = 0.37, *I* ^2^ = 5%). The results indicated that the raw incidence of adverse drug reactions was no significant differences in the control group than that in the intervention group [MD = 1.81, 95% CI (0.88–3.74), *p* = 0.11] (Figure 5).

##### 4.4.6.2 The details of adverse drug reactions

Reports of adverse drug reactions were few in the included studies, with six cases of gastrointestinal reaction, one case of fatigue, and five cases of nausea and one case of infection in the experimental group (Table 3).

**TABLE 3:** The details of reported adverse drug reactions.

Adverse reactions	Experimental (O. *sinensis* + dialysis)	Control (dialysis)
Gastrointestinal reaction	6	5
Dizziness	0	1
Fatigue	1	5
Drowsiness	0	2
Headache	0	2
Nausea	5	4
Infection	1	2
Hypotension	0	1

### 4.5 Subgroup analysis

#### 4.5.1 CRP

The meta analysis showed that the CRP levels significantly decreased in the intervention group compared with in the control group for patients treated by HD (11 studies, MD: 1.96, 95% CI [−3.17 to −0.75]; *I* ^2^ = 98%). For patients treated by PD, the CRP levels decreased in the intervention group compared with in the control group (4 studies, MD: −2.93, 95% CI [−4.88 to-0.98]; *I* ^2^ = 97%) (Supplementary Figure S2A).

In subgroup analyses of different *O. sinensis* preparations, the CRP levels significantly decreased in Jinshuibao capsule group compared with the control group (6 studies, MD: −0.68, 95% CI [−1.08 to −0.27]; *I* ^2^ = 30%). The CRP levels significantly decreased in the Bailing capsule group compared with the control group (15 studies, MD: −2.48, 95% CI [−3.70 to −1.26]; *I* ^2^ = 98%) (Supplementary Figure S3A).

In the subgroup analysis on the impact of intervention duration, the CRP levels significantly decreased in the intervention group compared with the control group (13 studies, MD: −1.95, 95% CI [−3.35 to −0.55]; *I* ^2^ = 98%) in the duration ≤12 weeks. The CRP levels significantly decreased in the intervention group compared with the control group in the duration>12 weeks (6 studies, MD: −2.64, 95% CI [−4.13 to −1.15]; *I* ^2^ = 97%) (Supplementary Figure S4A).

In the subgroup analysis of different doses, the CRP levels significantly decreased in the intervention group compared with the control group for patients taking 2–3 capsules (6 studies, MD: −3.32, 95% CI [−6.03 to −0.60; *I* ^2^ = 99%). The CRP levels significantly decreased in the intervention group compared with the control group for patients taking 4–6 capsules (12 studies, MD: −1.87, 95% CI [−2.93 to −0.82]; *I* ^2^ = 97%). No significant difference was found between the control and intervention group for patients taking >6 capsules [1 study, MD −0.27, 95% CI (−0.80, 0.26)] (Supplementary Figure S5A).

#### 4.5.2 ALB

In the subgroup analysis of different dialysis methods, the ALB levels significantly increased in the intervention group compared with the control group for patients treated by HD [12 studies, MD: 0.81, 95% CI (0.24 to 1.37); *I* ^2^ = 95%]. The ALB levels significantly increased in the intervention group compared with the control group for patients treated by PD [8 studies, MD: 0.81, 95% CI (0.24–1.38); *I* ^2^ = 91%] (Supplementary Figure S2B).

In subgroup analyses of different *O. sinensis* preparations, the ALB levels significantly increased in the Jinshuibao capsule group compared with the control group [6 studies, MD: 1.32, 95% CI (0.85 to 1.79)]. The ALB levels significantly increased in the Bailing capsule group compared with the control group [19 studies, MD: 0.78, 95% CI (0.36–1.20); *I* ^2^ = 94%] (Supplementary Figure S3B).

In the subgroup analysis of different intervention duration, the ALB levels significantly increased in the intervention group compared with the control group [14 studies, MD: 0.72, 95% CI (0.13 to 1.30); *I* ^2^ = 95%] in the duration ≤12 weeks; The ALB levels significantly increased in the intervention group compared with the control group during the study period >12 weeks [8 studies, MD: 0.73, 95% CI (0.38–1.08); *I* ^2^ = 75%] (Supplementary Figure S4B).

In the subgroup analysis of different doses of *O. sinensis* preparation, the ALB levels significantly increased in the intervention group compared with the control group for the patients taking 2–3 capsules [10 studies, MD: 0.66, 95% CI (0.34–0.99); *I* ^2^ = 76%]. The ALB levels significantly increased in the intervention group compared with the control group for the patients taking 4–6 capsules [10 studies, MD: 1.33, 95% CI (0.56–2.09); *I* ^2^ = 95%]; No significant difference was found in the ALB level between the two groups for the patients taking 4–6 capsules [3 studies, MD: 0.60, 95% CI (–0.17 to 1.38); *I* ^2^ =91%] (Supplementary Figure S5B).

#### 4.5.3 HGB

In the subgroup analysis of different dialysis methods, for patients treated by HD, the HGB levels significantly increased in the intervention group compared with the control group [12 studies, MD: 1.23, 95% CI (0.75–1.71); *I* ^2^ = 92%]. The HGB levels significantly increased in the intervention group compared with the control group for patients treated by PD [7 studies, MD: 0.95, 95% CI (0.41–1.49); *I* ^2^ = 88%] (Supplementary Figure S2C).

In subgroup analysis of different *O. sinensis* preparations, no significant differences were found in the HGB level between the Jinshuibao capsule group and the control group [6 studies, MD: 0.54, 95% CI (−0.02 to 1.10)]. The HGB levels significantly increased in the Bailing capsule group compared with the control group [16 studies, MD: 1.28, 95% CI (0.88–1.69); *I* ^2^ = 90%] (Supplementary Figure S3C).

In the subgroup analysis of the different intervention duration, the HGB levels significantly increased in the intervention group compared with the control group in the study period ≤12 weeks [14 studies, MD: 1.18, 95% CI (0.64–1.71), *I* ^2^ = 93%]. The HGB levels significantly increased in the intervention group compared with the control group during the study period > 12 weeks [8 studies, MD: 1.07, 95% CI (0.63–1.50), *I* ^2^ = 84%] (Supplementary Figure S4C).

In the subgroup analysis of different doses of *O. sinensis* preparation, the HGB levels significantly increased in the intervention group compared with the control group for patients taking 2–3 capsules [11 studies, MD: 1.29, 95% CI (0.83–1.76), *I* ^2^ = 90%]. In HGB levels, the intervention group was significantly increased compared with the control group for patients taking 4–6 capsules [9 studies, MD: 1.08, 95% CI (0.39–1.76), *I* ^2^ = 92%]. No significant found in the HGB level between the two groups for patients taking >6 capsules [2 studies, MD: 0.36, 95% CI (–0.01 to 0.72), *I* ^2^ = 0] (Supplementary Figure S5C).

#### 4.5.4 CREA

In the subgroup analysis of different dialysis methods, the CREA levels significantly decreased in the intervention group compared with the control group for patients treated by HD [13 studies, MD: −1.19, 95% CI (−1.70 to −0.68), *I* ^2^ = 94%]. The CREA levels significantly decreased in the intervention group compared with the control group for patients treated by PD [9 studies, MD –1.56, 95% CI (–2.52 to–0.59), *I* ^2^ = 96%] (Supplementary Figure S2D).

In the subgroup analysis of different *O. sinensis* preparations, the CREA levels significantly decreased in Jinshuibao capsule group compared with the control group [6 studies, MD: −1.26, 95% CI (−2.07 to −0.44), *I* ^2^ = 93%]. The CREA level was decreased in Bailing capsule intervention group compared with the control group [17 studies, MD: −1.35, 95% CI (−1.92 to −0.79), *I* ^2^ = 95%] (Supplementary Figure S3D).

In the subgroup analysis of different intervention duration, the CREA levels significantly decreased in the intervention group compared with the control group with the study period ≤12 weeks [15 studies, MD: −1.08, 95% CI (−1.52 to −0.64), *I* ^2^ = 93%]. The CREA levels significantly decreased in the intervention group compared with the control group in the study period > 12 weeks [8 studies, MD: −1.93, 95% CI (−3.27 to −0.59), *I* ^2^ = 97%] (Supplementary Figure S4D).

In the subgroup analysis of different doses of *O. sinensis* preparation, the CREA levels significantly decreased in the intervention group compared with the control group for patients taking 2–3 capsules [6 studies, MD: −2.08, 95% CI (−3.07 to −1.10), *I* ^2^ = 95%]. The CREA levels significantly decreased in the intervention group compared with the control group for patients taking 4–6 capsules [14 studies, MD: −1.06, 95% CI (−1.63 to −0.48), *I* ^2^ = 94%]. No significant difference in the CREA level was found in the intervention group than in the control group for patients taking >6 capsules [3 studies, MD: −1.01, 95% CI (−2.11 to 0.09), *I* ^2^ = 94%] (Supplementary Figure S5D).

#### 4.5.5 BUN

In the subgroup analysis of different dialysis methods, the BUN levels significantly decreased in the intervention group compared with the control group for patients treated by HD [13 studies, MD: −1.69, 95% CI (−2.34 to −1.03); *I* ^2^ = 96%]. The BUN levels significantly decreased in the intervention group compared with the control group for patients treated by PD [8 studies, MD: –1.38, 95% CI (−2.16 to −0.60); *I* ^2^ = 94%] (Supplementary Figure S2E).

In the subgroup analysis of different *O. sinensis* preparations, the BUN levels significantly decreased in Jinshuibao capsule group compared with the control group [6 studies, MD −1.36, 95% CI (−1.99 to −0.73), *I* ^2^ = 89%]. The BUN levels significantly decreased in Bailing capsule group compared with the control group [14 studies, MD: −1.57, 95% CI (–2.27 to −0.86), *I* ^2^ = 96%] (Supplementary Figure S3E).

In the subgroup analysis of different intervention duration, the BUN levels significantly decreased in the intervention group compared with the control group within the study period ≤12 weeks [15 studies, MD: −1.51, 95% CI (−2.11 to −0.91), *I* ^2^ = 95%]; The BUN levels significantly decreased in the intervention group compared with the control group in the study period> 12 weeks [7 studies, MD: −1.73, 95% CI (−2.74 to −0.72), *I* ^2^ = 95%] (Supplementary Figure S4E).

In the subgroup analysis of different doses of *O. sinensis* preparation, the BUN levels significantly decreased in the intervention group compared with the control group for patients taking 2–3 capsules [6 studies, MD: −1.78, 95% CI (−2.78 to −0.77), *I* ^2^ = 95%]. The BUN levels significantly decreased in the intervention group compared with the control group for patients taking the 4–6 capsules [13 studies, MD: −1.64, 95% CI (−2.39 to −0.89), *I* ^2^ = 96%]; The BUN levels significantly decreased in the intervention group compared with the control group for patients taking >6 capsules [3 studies, MD: −0.95, 95% CI (−1.65 to −0.25), *I* ^2^ = 87%] (Supplementary Figure S5E). Details of the above subgroup analysis are shown in the table below (Table 4).

**TABLE 4:** Subgroups analysis of outcomes.

Outcome	Subgroup		N	MD/SMD (95% CI)	I^2^ (%)
CRP	Different dialysis methods	Hemodialysis	11	−1.96 [-3.17, −0.75]	98
		Peritoneal dialysis	4	−2.93 [-4.88, −0.98]	97
	Different interventions	Jinshuibao capsule	6	−0.68 [-1.08, −0.27]	30
		Bailing capsule	15	−2.48 [-3.70, −1.26]	98
	Different intervention duration	T ≤ 12 weeks	13	−1.95 [-3.35, −0.55]	98
		T > 12 weeks	6	−2.64 [-4.13, −1.15]	97
	Different doses of drugs	2–3 capsules	6	−3.32 [-6.03, −0.60]	99
		4–6 capsules	12	−1.87 [-2.93, −0.82]	97
		>6 capsules	1	−0.27 [-0.80, 0.26]	-
ALB	Different dialysis methods	Hemodialysis	12	0.81 [0.24, 1.37]	95
		Peritoneal dialysis	8	0.81 [0.24, 1.38]	91
	Different interventions	Jinshuibao capsule	6	1.32 [0.85, 1.79]	-
		Bailing capsule	19	0.78 [0.36, 1.20]	94
	Different intervention duration	T ≤ 12 weeks	14	0.72 [0.13, 1.30]	95
		T > 12 weeks	8	0.73 [0.38, 1.08]	93
	Different doses of drugs	2–3 capsules	10	0.66 [0.34, 0.99]	76
		4–6 capsules	10	1.33 [0.56, 2.09]	95
		>6 capsules	3	0.60 [-0.17, 1.38]	88
HGB	Different dialysis methods	Hemodialysis	12	1.23 [0.75, 1.71]	92
		Peritoneal dialysis	7	0.95 [0.41, 1.49]	88
	Different interventions	Jinshuibao Capsule	6	0.54 [-0.02, 1.10]	82
		Bailing Capsule	16	1.28 [0.88, 1.69]	90
	Different intervention duration	T ≤ 12 weeks	14	1.18 [0.64, 1.71]	93
		T > 12 weeks	8	1.07 [0.63, 1.50]	84
	Different doses of drugs	2–3 capsules	11	1.29 [0.83, 1.76]	90
		4–6 capsules	9	1.08 [0.39, 1.76]	92
		>6 capsules	2	0.36 [-0.01, 0.72]	0
CREA	Different dialysis methods	Hemodialysis	13	−1.19 [-1.70, −0.68]	94
		Peritoneal dialysis	9	−1.56 [-2.52, −0.59]	96
	Different interventions	Jinshuibao capsule	6	−1.26 [-2.07, −0.44]	93
		Bailing capsule	17	−1.35 [-1.92, −0.79]	95
	Different intervention durations	T ≤ 12 weeks	15	−1.08 [-1.52, −0.64]	93
		T > 12 weeks	8	−1.93 [-3.27, −0.59]	97
	Different doses of drugs	2–3 capsules	6	−2.08 [-3.07, −1.10]	95
		4–6 capsules	14	−1.06 [-1.63, −0.48]	94
		>6 capsules	3	−1.01 [-2.11, 0.09]	94
BUN	Different dialysis methods	Hemodialysis	13	−1.69 [-2.34, −1.03]	96
		Peritoneal dialysis	8	−1.38 [-2.16, −0.60]	94
	Different interventions	Jinshuibao Capsule	6	−1.36 [-1.99, −0.73]	89
		Bailing Capsule	14	−1.57 [-2.27, −0.86]	96
	Different intervention duration	T ≤ 12 weeks	15	−1.51 [-2.11, −0.91]	95
		T > 12 weeks	7	−1.73 [-2.74, −0.72]	95
	Different doses of drugs	2–3 capsules	6	−1.78 [-2.78, −0.77]	95
		4–6 capsules	13	−1.64 [-2.39, −0.89]	96
		>6 capsules	3	−0.95 [-1.65, −0.25]	87

N, number of studies.

### 4.6 Sensitivity analysis

We performed a sensitivity analysis to assess the robustness of the results and found good robustness after excluding the literature one by one.

### 4.7 Publication bias

**FIGURE 6:**
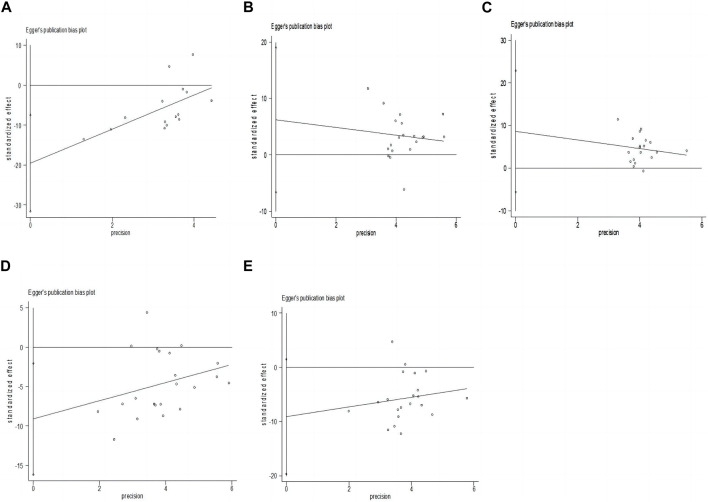
Funnel plot of publication bias on five essential outcomes. **(A)** CRP; **(B)** ALB; **(C)** HGB; **(D)** CREA; **(E)** BUN.

We performed an Egger’s test on five essential outcomes to observe the publication bias, and CRP (*p* = 0.002 < 0.05), CREA (*p* = 0.019 < 0.05), BUN(*p* = 0.025 < 0.05) were showed publication bias, and ALB, HGB *p* values were >0.05, with no publication bias. After assessing the impact of publication bias on the results with trim-and-fill analyses, the results were found to be reliable (Figure 6).

### 4.8 Quality of the evidence

GRADE was used to assess the quality of outcome evidence for all studies. All outcomes were rated as low or very low according to the GRADE criteria because of serious imprecision and large heterogeneity in findings, and indirectness due to a mix of different interventions and comparators (Table 5).

**TABLE 5:** Summary of findings table.

**Artificial cordyceps preparation compared to dialysis + conventional treatment for dialysis**							
Patient or population: Dialysis Settings Intervention: Artificial cordyceps preparation Comparison: Dialysis + conventional treatment							

Outcomes	Illustrative comparative risks* (95% CI)			Relative effect (95% CI)	No of Participants (studies)	Quality of the evidence (GRADE)	Comments
	Assumed risk	Corresponding risk					
	Dialysis + conventional treatment	Artificial Cordyceps Preparation					
C-RP		The mean c-rp in the intervention groups was 2.22 standard deviations lower (3.24–1.2 lower)			1,191 (15 studies)	⊕⊝⊝⊝ very low^1^	
ALB		The mean alb in the intervention groups was 0.81 standard deviations lower (1.21–0.41 lower)			1765 (20 studies)	⊕⊕⊝⊝ low^1^	
HGB		The mean hgb in the intervention groups was 1.00 standard deviations lower (1.43–0.57 lower)			1,568 (19 studies)	⊕⊕⊝⊝ low^1^	
CREA		The mean crea in the intervention groups was 1.33 standard deviations higher (0.87–1.79 higher)			1840 (22 studies)	⊕⊝⊝⊝ very low^1^	
BUN		The mean bun in the intervention groups was 1.57 standard deviations higher (1.07–2.07 higher)			1805 (21 studies)	⊕⊕⊝⊝ low^1^	
Adverse Reactions	Study population			OR 1.81 (0.88–3.74)	346 (4 studies)	⊕⊕⊝⊝ low^1^	
	8 per 100	13 per 100 (7–23)					
	Moderate						
		1					
*The basis for the **assumed risk** (e.g., the median control group risk across studies) is provided in footnotes. The **corresponding risk** (and its 95% confidence interval) is based on the assumed risk in the comparison group and the **relative effect** of the intervention (and its 95% CI).							
**CI:** confidence interval; **OR:** Odds ratio.							
GRADE working group grades of evidence. **High quality:** Further research is very unlikely to change our confidence in the estimate of effect. **Moderate quality:** Further research is likely to have an important impact on our confidence in the estimate of effect and may change the estimate. **Low quality:** Further research is very likely to have an important impact on our confidence in the estimate of effect and is likely to change the estimate. **Very low quality:** We are very uncertain about the estimate.							
^1^All studies in this analysis had unclear methods for allocation concealment. ^2^95% CI, is wide and consistent with both moderate harm and benefit. ^3^There was substantial heterogeneity in the findings of available studies. ^4^All outcomes in this analysis were not clearly blinded in all studies.							

## 5 Discussion

This review included related RCTs to assess the effects and safety of *O. sinensis* preparations in adjuvant treatment for patients undergoing dialysis. Alternative outcomes were used due to the lack of reports of the results from the COMET core outcome index set. After meta-analysis, the results showed that *O. sinensis* preparations could reduce the CREA, BUN, and CRP levels and increase the ALB and HGB levels. Considering the clinical heterogeneity and evidence quality, there are no high-quality evidence to support the use of *O. sinensis* preparations in adjuvant treatment for patients undergoing dialysis and their harms are under-reported.

From 1990 to 2017, the incidence of dialysis increased by 43.1% with the development of dialysis technology (GBD Chronic Kidney Disease Collaboration, 2020). Approximately 89% of patients undergoing dialysis are treated with HD worldwide, while a minority are treated with PD (Al. and Al). The global dialysis population is proliferating, especially in low- and middle-income countries (Bello et al., 2017); however, many people lack access to kidney replacement therapy, and millions of people die of kidney failure annually worldwide, often without supportive care (Himmelfarb et al., 2020). Thus, new approaches and dialysis modalities that are accessible and offer improved patient outcomes urgently need to be developed.

Chinese caterpillar fungus, or Dong Chong Xia Cao (winter worm summer grass) in Chinese or Tochukaso in Japanese, has been used in China for over 700 years, mainly as a tonic for nourishing the lungs and kidneys (Dong and Yao, 2008). Modern pharmacological studies have shown its therapeutic effect on various diseases and conditions such as the kidneys (Ding et al., 2011; Zhang et al., 2011) as well as on other diseases (Yue et al., 2013b). However, the output of natural *O. sinensis* cannot fully meet the demands of medical use due to the scarcity of resources and high price, which drives many types of artificial cultivation to make *O. sinensis* a more affordable material for its use (Qian et al., 2019; Wu et al., 2020; Wang et al., 2022). The highest cordycepin production can be obtained in surface liquid culture using the *C. militaris* mutant.

The artificial cultivation of *C. militaris* produces cordycepin. It has a similar pharmacological activity to *O. sinensis*, which is more accessible; also, multiproduct batch manufacturing has been achieved (Sari et al., 2016; Yue et al., 2013a). This review aimed to assess the role of *O. sinensis* preparation in the adjuvant treatment for both patients undergoing HD and those undergoing PD. This was the first systematic review to evaluate the efficacy and safety of *O. sinensis* preparation in adjuvant treatment for two kinds of patients undergoing dialysis (HD and PD). Although a systematic review and meta-analysis of hemodialysis patients was published in 2019, (Bee and Zoriah, 2019), our study, differed greatly from this review. Compared with the previous systematic review, we have some difference in the following aspects. First, our study extended the study population and covered a wider range of subjects, including not only patients undergoing HD but also those undergoing PD. Second, a core outcome set (COS) is the minimum that should be measured and reported in all clinical trials of a specific condition, which also helps streamline the systematic reviewing process (Clarke and Williamson, 2016). Therefore, we searched the COMET database and referred to the dialysis-COS to set the primary and secondary outcomes for our review. Third, we compared the patients with HD to those with PD in response to *O. sinens*is (Li et al., 2018; Pan, 2019). Altogether, it has been 6 years since the literature search in the last systematic review, and since then, new research evidence has increased. We included latest related studies in recent 6 years, by adding 23 additional studies, and the number of included patients increased to 2,914. Accordingly, new clinical research quesionss with different PICOS have emerged, urging us to carry out a new systematic review. We hope that this systematic review can provide evidence of efficacy and safety for patients undergoing dialysis when using *O. sinensis* preparation.

### 5.1 Limitations

This review was conducted according to a pre-specified protocol and used a highly sensitive search strategy. Two review authors conducted an electronic database search independently and accorded to the evidence certainty for analyzing the results. However, it had several limitations. First, it had restrictions on language, affecting its comprehensiveness. Second, some studies had risk of detection and performance bias due to the lack of blinding. Third, we used COMET outcomes, but only a few studies reported the outcomes. We included other outcomes not directly related to ESRD in this review due to the lack of data, which downgraded the level of evidence. Fourth, the condition of patients in the dialysis period had a certain complexity, and the simultaneous existence of the primary disease and comorbidities led to great clinical heterogeneity and affected our judgment of the results. However, we did not perform a subgroup analysis of patients with different primary diseases or comorbidity due to the lack of study reports, which might account for a risk due to inconsistency.

## 6 Conclusion

In conclusion, *O. sinensis* can serve as an adjuvant treatment for patients undergoing dialysis by improving patient renal function, malnutrition, and microinflammation. However, few studies reported clinically relevant outcomes and the methodological quality of the included studies were generally low. Therefore, to using COMET outcomes in trials and providing more reliable evidence through high-quality RCTs are necessary.

## Data Availability

The original contributions presented in the study are included in the article/Supplementary Material, further inquiries can be directed to the corresponding authors.
