# Supplementary material for: Effects and safety of Ophiocordyceps sinensis preparation in the adjuvant treatment for dialysis patients: a systematic review and meta-analysis
Source: Front Pharmacol. 2024 Jul 19;15:1360997. doi: 10.3389/fphar.2024.1360997 (PMC11294943; doi:10.3389/fphar.2024.1360997)
Supplement: Supplementary file 1 [file DataSheet2.pdf]

Study or Subgroup	Experimental Mean	Experimental SD	Experimental Total	Control Mean	Control SD	Control Total	Weight	Std. Mean Difference IV, Random, 95% CI
**2.1.1 hemodialysis**								
Chen 2017	3.15	1.06	25	4.85	1.62	25	6.7%	-1.22 [-1.83, -0.61]
Cui 2020	8.92	2.98	66	4.12	1.01	34	6.8%	1.91 [1.41, 2.40]
Gao 2019	4.56	0.87	41	6.73	1.08	41	6.7%	-2.19 [-2.74, -1.64]
Huang 2018	3.56	0.3	50	6.62	0.29	50	6.0%	-10.29 [-11.80, -8.78]
Sun 2011	2.4	1.4	69	9.3	3.5	33	6.7%	-2.99 [-3.58, -2.40]
Wang2023	6.72	1.48	30	7.39	1.46	30	6.8%	-0.45 [-0.96, 0.06]
Wangb 2019	5.1	1.2	28	9.7	1.4	28	6.6%	-3.48 [-4.33, -2.63]
Wu 2012	7.5	1.99	42	5.01	1.37	21	6.7%	1.36 [0.78, 1.94]
Zhang2022	6.47	1.53	44	8.12	2.21	42	6.8%	-0.86 [-1.31, -0.42]
Zhao2022	1.28	0.57	40	2.79	0.88	40	6.8%	-2.02 [-2.56, -1.47]
Zhu 2019	6.42	1.34	45	9.92	1.63	45	6.8%	-2.33 [-2.87, -1.79]
**Subtotal (95% CI)**			**480**			**389**	**73.3%**	**-1.96 [-3.17, -0.75]**
Heterogeneity: Tau² = 4.06; Chi² = 477.50, df = 10 (P < 0.00001); I² = 98%								
Test for overall effect: Z = 3.18 (P = 0.001)								
**2.1.2 peritoneal dialysis**								
Liu 2014	6.2	2.3	28	6.8	2.1	28	6.8%	-0.27 [-0.80, 0.26]
Sun 2012	3.1	1.5	53	9.8	2.5	49	6.7%	-3.26 [-3.86, -2.66]
Zhanga 2020	6.3	0.83	43	9.18	1.21	43	6.7%	-2.75 [-3.35, -2.15]
Zhu 2020	2.06	0.34	39	5.19	0.71	39	6.5%	-5.57 [-6.57, -4.57]
**Subtotal (95% CI)**			**163**			**159**	**26.7%**	**-2.93 [-4.88, -0.98]**
Heterogeneity: Tau² = 3.82; Chi² = 110.18, df = 3 (P < 0.00001); I² = 97%								
Test for overall effect: Z = 2.95 (P = 0.003)								
**Total (95% CI)**			**643**			**548**	**100.0%**	**-2.22 [-3.24, -1.20]**
Heterogeneity: Tau² = 3.97; Chi² = 628.64, df = 14 (P < 0.00001); I² = 98%								
Test for overall effect: Z = 4.25 (P < 0.0001)								
Test for subgroup differences: Chi² = 0.68, df = 1 (P = 0.41), I² = 0%								

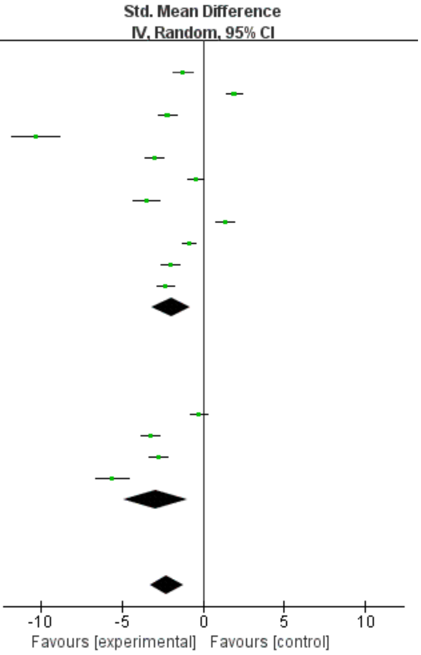

Fig.S2A

Study or Subgroup	Experimental Mean	Experimental SD	Experimental Total	Control Mean	Control SD	Control Total	Weight	Std. Mean Difference IV, Random, 95% CI
**2.2.1 hemodialysis**								
Cui 2020	35.42	4.68	66	42.41	5.18	34	5.0%	-1.43 [-1.89, -0.97]
Gao 2019	43.25	1.45	41	40.76	1.8	41	5.0%	1.51 [1.02, 2.00]
Huang 2018	39.65	6.56	50	35.62	6.23	50	5.1%	0.63 [0.22, 1.03]
Li2023	44.82	7.28	65	41.19	5.31	65	5.2%	0.57 [0.22, 0.92]
Sun 2011	36.9	10.8	69	29.8	7.5	33	5.1%	0.71 [0.29, 1.14]
Wanga 2019	42.36	2.68	75	38.46	3.27	75	5.2%	1.30 [0.94, 1.65]
Wu 2012	32.56	11.38	42	33.12	9.66	21	4.9%	-0.05 [-0.57, 0.47]
Yang 2019	39.12	5.98	36	34.59	6.02	36	5.0%	0.75 [0.27, 1.23]
Zeng2023	36.29	1.25	54	31.14	1.42	54	4.7%	3.82 [3.18, 4.46]
Zhang2022	41.19	3.85	44	35.49	4.67	42	5.0%	1.32 [0.85, 1.79]
Zhao2022	41.17	5.46	40	40.03	5.21	40	5.0%	0.21 [-0.23, 0.65]
Zhu 2019	42.19	4.83	45	39.73	4.91	45	5.1%	0.50 [0.08, 0.92]
**Subtotal (95% CI)**			**627**			**536**	**60.3%**	**0.81 [0.24, 1.37]**
Heterogeneity: Tau² = 0.94; Chi² = 215.37, df = 11 (P < 0.00001); I² = 95%								
Test for overall effect: Z = 2.81 (P = 0.005)								
**2.2.2 peritoneal dialysis**								
Liu 2014	41.9	2.7	28	41.2	2.3	28	4.9%	0.28 [-0.25, 0.80]
Sun 2012	36	10	53	30	8	49	5.1%	0.65 [0.26, 1.05]
Tian 2014	29.51	4.97	30	30.16	5.81	28	4.9%	-0.12 [-0.63, 0.40]
Tian 2021	35.31	4.41	30	33.11	5.27	30	4.9%	0.45 [-0.07, 0.96]
Wang 2016	42.1	3.2	30	41.5	3.4	30	4.9%	0.18 [-0.33, 0.69]
Ying 2018	42.31	1.78	47	39.46	1.51	47	5.0%	1.71 [1.24, 2.19]
Zhangb 2020	38.43	3.08	47	31.61	2.17	47	4.9%	2.54 [1.99, 3.09]
Zhu 2020	45.52	4.67	39	41.68	4.73	39	5.0%	0.81 [0.35, 1.27]
**Subtotal (95% CI)**			**304**			**298**	**39.7%**	**0.81 [0.24, 1.38]**
Heterogeneity: Tau² = 0.62; Chi² = 76.86, df = 7 (P < 0.00001); I² = 91%								
Test for overall effect: Z = 2.77 (P = 0.006)								
**Total (95% CI)**			**931**			**834**	**100.0%**	**0.81 [0.41, 1.21]**
Heterogeneity: Tau² = 0.78; Chi² = 292.79, df = 19 (P < 0.00001); I² = 94%								
Test for overall effect: Z = 3.94 (P < 0.0001)								
Test for subgroup differences: Chi² = 0.00, df = 1 (P = 0.99), I² = 0%								

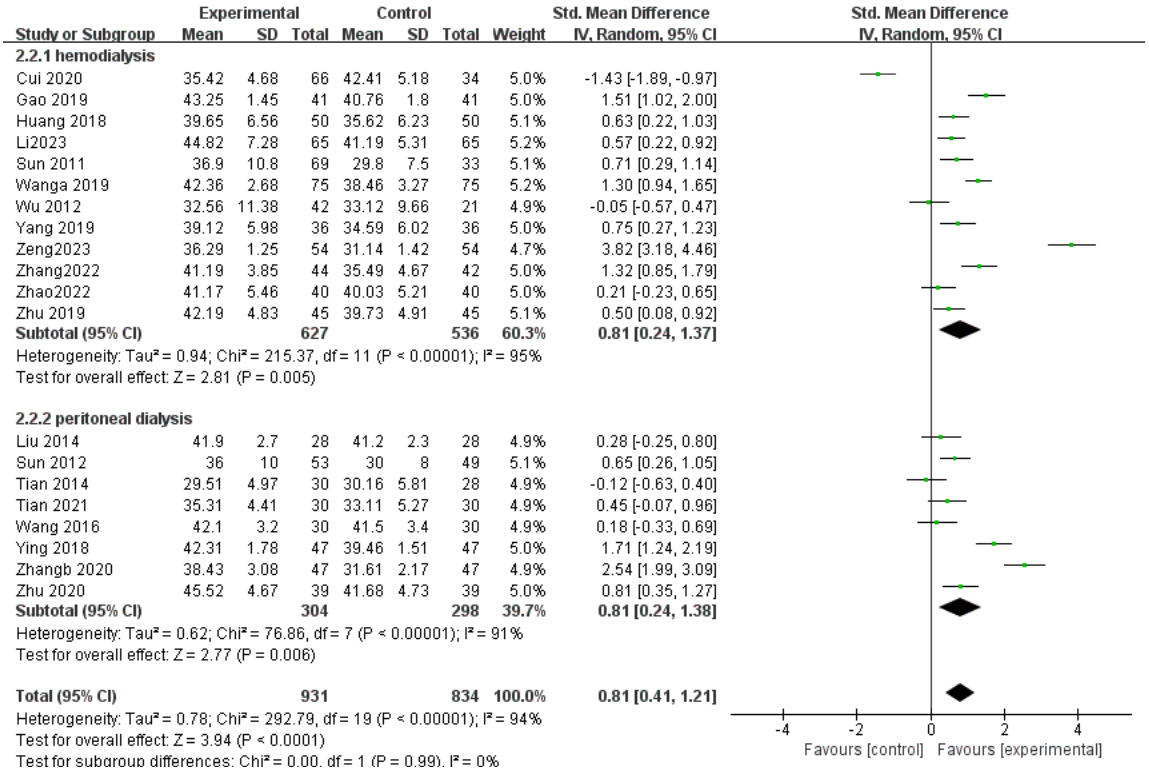

Fig.S2B

	Experimental			Control				Std. Mean Difference
**Study or Subgroup**	**Mean**	**SD**	**Total**	**Mean**	**SD**	**Total**	**Weight**	**IV, Random, 95% CI**
**2.3.1 hemodialysis**								
Gao 2019	108.42	9.36	41	94.65	4.78	41	5.2%	1.84 [1.32, 2.36]
He 2021	106.5	9.44	38	95.69	8.31	38	5.3%	1.20 [0.71, 1.69]
Li2023	92.83	8.24	65	86.1	9.75	65	5.5%	0.74 [0.39, 1.10]
Lin2024	115.53	12.53	40	108.64	11.44	40	5.4%	0.57 [0.12, 1.02]
Ling 2021	102.35	7.83	41	92.67	7.65	41	5.3%	1.24 [0.76, 1.71]
Ren 2021	147.91	6.45	55	125.57	6.41	55	5.0%	3.45 [2.85, 4.05]
Sun 2011	123.1	19	69	97.4	9.2	33	5.3%	1.54 [1.08, 2.01]
Wang2023	89.07	9.26	30	83.36	12.3	30	5.2%	0.52 [0.00, 1.03]
Yang 2019	93.74	11.28	36	84.01	9.83	36	5.3%	0.91 [0.42, 1.40]
Ye 2012	104.69	12.46	34	106.72	13.09	34	5.3%	-0.16 [-0.63, 0.32]
Zeng2023	109.38	6.24	54	95.21	6.28	54	5.3%	2.25 [1.76, 2.73]
Zhu 2019	103.64	11.09	45	94.86	10.32	45	5.4%	0.81 [0.38, 1.24]
**Subtotal (95% CI)**			**548**			**512**	**63.4%**	**1.23 [0.75, 1.71]**
Heterogeneity: Tau² = 0.65; Chi² = 135.96, df = 11 (P < 0.00001); I² = 92%								
Test for overall effect: Z = 5.05 (P < 0.00001)								
**2.3.2 peritoneal dialysis**								
Liu 2014	102.4	8.3	28	98.7	9.4	28	5.2%	0.41 [-0.12, 0.94]
Sun 2012	119	12	53	96	9	49	5.3%	2.14 [1.65, 2.63]
Tian 2014	89.4	11.61	30	88.16	12.64	28	5.2%	0.10 [-0.41, 0.62]
Tian 2021	89.2	11.56	30	77.18	12.24	30	5.2%	1.00 [0.46, 1.54]
Wang 2016	102.6	8.5	30	99.8	9.6	30	5.2%	0.30 [-0.20, 0.81]
Ying 2018	98.42	3.25	47	94.25	2.72	47	5.3%	1.38 [0.93, 1.83]
Zhu 2020	108.67	8.51	39	97.85	8.13	39	5.3%	1.29 [0.80, 1.78]
**Subtotal (95% CI)**			**257**			**251**	**36.6%**	**0.95 [0.41, 1.49]**
Heterogeneity: Tau² = 0.46; Chi² = 48.37, df = 6 (P < 0.00001); I² = 88%								
Test for overall effect: Z = 3.46 (P = 0.0005)								
**Total (95% CI)**			**805**			**763**	**100.0%**	**1.13 [0.77, 1.48]**
Heterogeneity: Tau² = 0.56; Chi² = 185.82, df = 18 (P < 0.00001); I² = 90%								
Test for overall effect: Z = 6.23 (P < 0.00001)								
Test for subgroup differences: Chi² = 0.58, df = 1 (P = 0.44), I² = 0%								

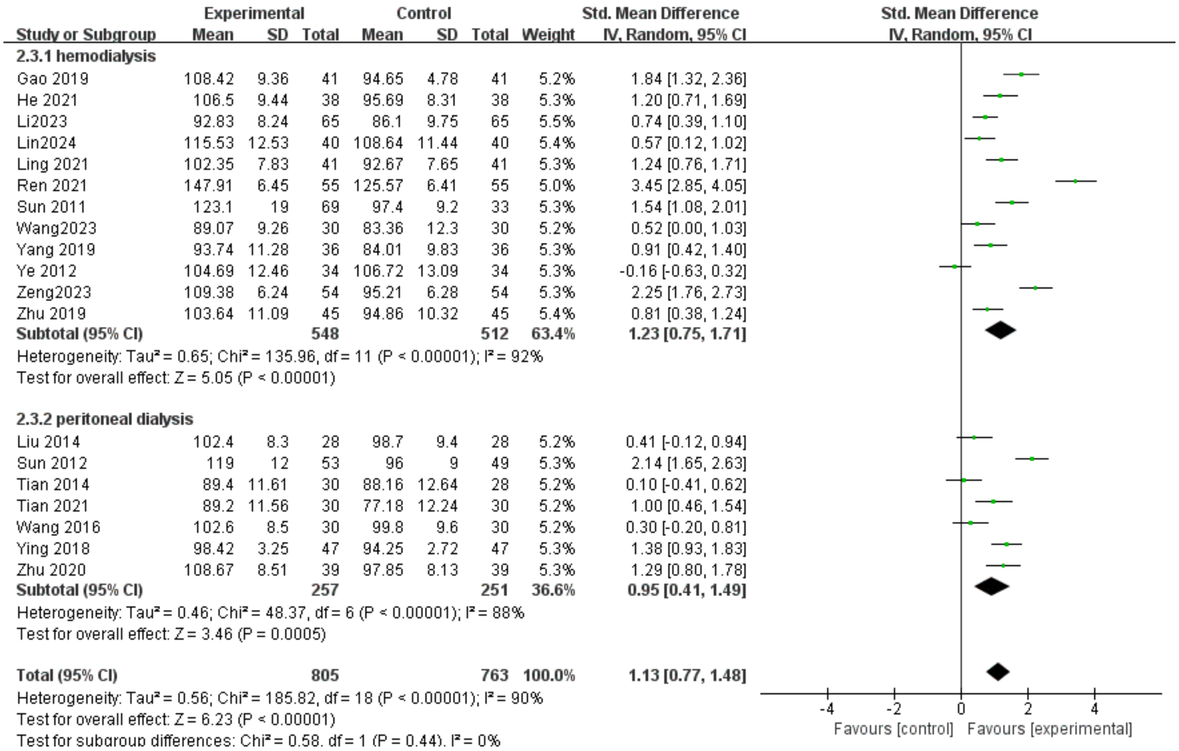

Fig.S2C

	Experimental			Control				Std. Mean Difference
Study or Subgroup	Mean	SD	Total	Mean	SD	Total	Weight	IV, Random, 95% CI
**2.4.1 hemodialysis**								
Gao 2019	175.52	16.85	41	234.51	23.27	41	4.5%	-2.88 [-3.50, -2.25]
Li2023	324.75	84.6	65	385.4	93.47	65	4.7%	-0.68 [-1.03, -0.32]
Lin2024	315.44	25.66	40	362.18	21.35	40	4.6%	-1.96 [-2.50, -1.42]
Ling 2021	248.25	22.34	41	301.64	30.47	41	4.6%	-1.98 [-2.51, -1.45]
Ren 2021	287.89	21.14	55	325.41	21.12	55	4.7%	-1.76 [-2.21, -1.32]
Tang 2024	385.73	102.62	63	423.64	103.55	63	4.7%	-0.37 [-0.72, -0.01]
Wanga 2019	832.52	84.25	75	902.35	96.57	75	4.7%	-0.77 [-1.10, -0.43]
Wangb 2019	697.5	53.1	28	849.7	60.3	28	4.3%	-2.64 [-3.37, -1.91]
Wu 2012	783.9	77.06	42	694.54	52.33	21	4.5%	1.26 [0.69, 1.83]
Ye 2012	575.69	29.97	34	581.27	30.21	34	4.6%	-0.18 [-0.66, 0.29]
Zeng2023	831.24	61.39	54	892.53	55.1	54	4.7%	-1.04 [-1.45, -0.64]
Zhang2022	157.95	12.47	44	188.76	19.68	42	4.6%	-1.86 [-2.37, -1.35]
Zhao2022	451.46	51.24	40	497.65	59.38	40	4.6%	-0.82 [-1.28, -0.37]
**Subtotal (95% CI)**			**622**			**599**	**59.9%**	**-1.19 [-1.70, -0.68]**
Heterogeneity: Tau² = 0.81; Chi² = 191.43, df = 12 (P < 0.00001); I² = 94%								
Test for overall effect: Z = 4.60 (P < 0.00001)								
**2.4.2 peritoneal dialysis**								
Li 2021	604.3	22.42	25	697.88	22.25	25	3.9%	-4.12 [-5.13, -3.12]
Liu 2014	916.8	102.7	28	923.3	107.4	28	4.6%	-0.06 [-0.58, 0.46]
Tian 2014	945.76	311.54	30	988.94	321.58	28	4.6%	-0.13 [-0.65, 0.38]
Tian 2021	341.4	108.41	30	660.71	185.27	30	4.5%	-2.08 [-2.71, -1.44]
Wang 2018	765	68	40	762	54	40	4.7%	0.05 [-0.39, 0.49]
Xie 2019	314.5	18.5	50	357.4	20.1	50	4.6%	-2.20 [-2.70, -1.70]
Yao 2016	546.58	109.09	17	541.67	114.78	18	4.4%	0.04 [-0.62, 0.71]
Ying 2018	845.29	7.56	47	885.13	8.98	47	4.2%	-4.76 [-5.56, -3.96]
Zhanga 2020	453.77	48.76	43	510.33	55.45	43	4.6%	-1.07 [-1.53, -0.62]
**Subtotal (95% CI)**			**310**			**309**	**40.1%**	**-1.56 [-2.52, -0.59]**
Heterogeneity: Tau² = 2.09; Chi² = 208.18, df = 8 (P < 0.00001); I² = 96%								
Test for overall effect: Z = 3.15 (P = 0.002)								
**Total (95% CI)**			**932**			**908**	**100.0%**	**-1.33 [-1.79, -0.87]**
Heterogeneity: Tau² = 1.13; Chi² = 399.90, df = 21 (P < 0.00001); I² = 95%								
Test for overall effect: Z = 5.66 (P < 0.00001)								
Test for subgroup differences: Chi² = 0.43, df = 1 (P = 0.51), I² = 0%								

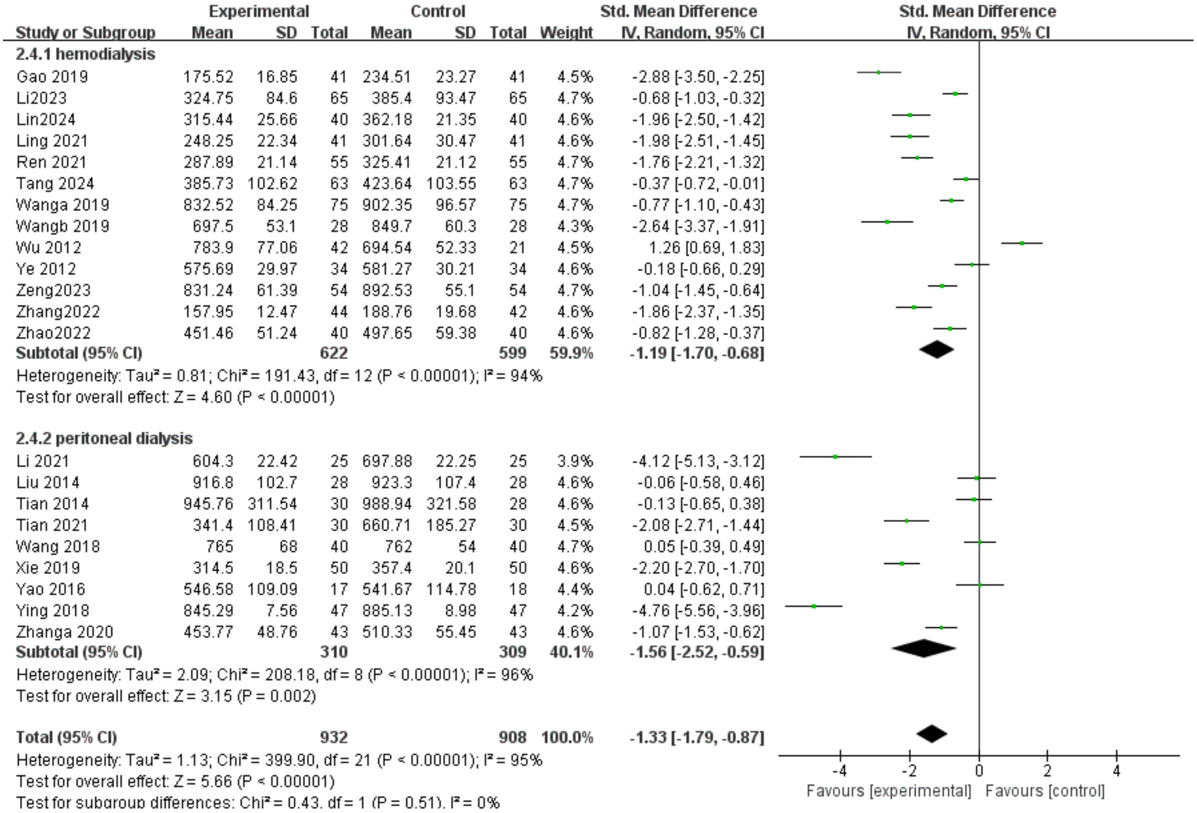

Fig.S2D

Study or Subgroup	Experimental Mean	Experimental SD	Experimental Total	Control Mean	Control SD	Control Total	Weight	Std. Mean Difference IV, Random, 95% CI
**2.5.1 hemodialysis**								
Gao 2019	13.26	2.87	41	18.98	2.33	41	4.8%	-2.17 [-2.72, -1.62]
Li2023	12.15	2.96	65	19.75	1.26	65	4.8%	-3.32 [-3.86, -2.79]
Lin2024	14.27	2.37	40	16.15	1.18	40	4.8%	-0.99 [-1.46, -0.53]
Ling 2021	11.33	2.05	41	15.78	2.34	41	4.8%	-2.00 [-2.54, -1.47]
Ren 2021	12.84	1.69	55	18.71	1.62	55	4.7%	-3.52 [-4.12, -2.92]
Tang 2024	13.82	2.19	63	17.94	2.21	63	4.9%	-1.86 [-2.28, -1.44]
Wanga 2019	21.31	3.28	75	25.14	4.37	75	4.9%	-0.99 [-1.33, -0.65]
Wangb 2019	16.1	3.8	28	28.9	7.3	28	4.6%	-2.17 [-2.84, -1.50]
Wu 2012	24.72	7.41	42	15.77	3.81	21	4.7%	1.37 [0.79, 1.95]
Ye 2012	21.63	3.19	34	22.57	3.91	34	4.8%	-0.26 [-0.74, 0.22]
Zeng2023	22.43	1.2	54	26.38	1.31	54	4.7%	-3.12 [-3.69, -2.55]
Zhang2022	15.54	2.25	44	19.69	2.63	42	4.8%	-1.68 [-2.18, -1.19]
Zhao2022	11.87	2.35	40	15.21	2.81	40	4.8%	-1.28 [-1.76, -0.79]
**Subtotal (95% CI)**			**622**			**599**	**62.2%**	**-1.69 [-2.34, -1.03]**
Heterogeneity: Tau² = 1.38; Chi² = 268.71, df = 12 (P < 0.00001); I² = 96%								
Test for overall effect: Z = 5.05 (P < 0.00001)								
**2.5.2 peritoneal dialysis**								
Li 2021	21.5	3.74	25	38.74	4.71	25	4.2%	-3.99 [-4.98, -3.00]
Liu 2014	26.3	5.2	28	27.4	5.1	28	4.8%	-0.21 [-0.74, 0.31]
Tian 2014	18.21	7.82	30	17.13	6.43	28	4.8%	0.15 [-0.37, 0.66]
Tian 2021	14.37	4.6	30	25.45	7.24	30	4.7%	-1.80 [-2.41, -1.20]
Wang 2018	26.7	6.7	40	27.6	4.3	40	4.9%	-0.16 [-0.60, 0.28]
Xie 2019	13.7	2.3	50	17.6	2.5	50	4.8%	-1.61 [-2.06, -1.16]
Ying 2018	24.21	0.87	47	26.65	1.04	47	4.8%	-2.52 [-3.07, -1.98]
Zhanga 2020	12.18	1.96	43	15.01	2.46	43	4.8%	-1.26 [-1.73, -0.80]
**Subtotal (95% CI)**			**293**			**291**	**37.8%**	**-1.38 [-2.16, -0.60]**
Heterogeneity: Tau² = 1.18; Chi² = 120.76, df = 7 (P < 0.00001); I² = 94%								
Test for overall effect: Z = 3.47 (P = 0.0005)								
**Total (95% CI)**			**915**			**890**	**100.0%**	**-1.57 [-2.07, -1.07]**
Heterogeneity: Tau² = 1.29; Chi² = 404.94, df = 20 (P < 0.00001); I² = 95%								
Test for overall effect: Z = 6.16 (P < 0.00001)								
Test for subgroup differences: Chi² = 0.35, df = 1 (P = 0.56), I² = 0%								

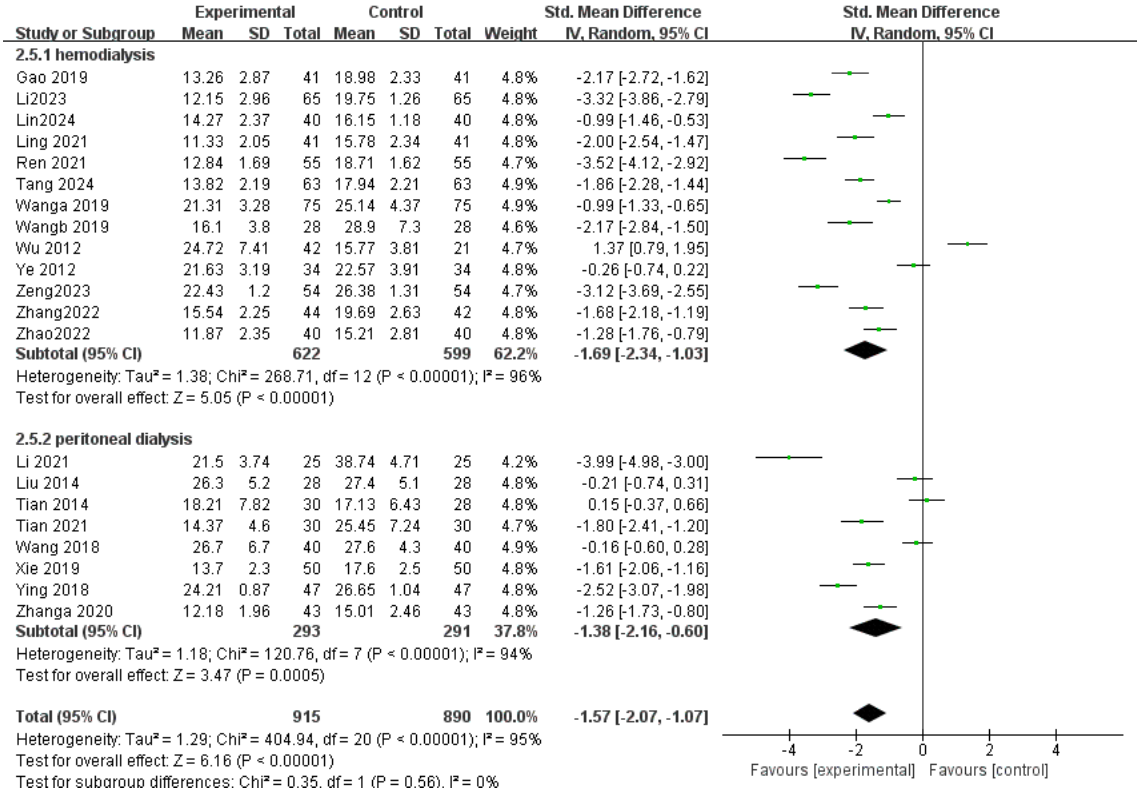

Fig.S2E

Study or Subgroup	Experimental Mean	Experimental SD	Experimental Total	Control Mean	Control SD	Control Total	Weight	Std. Mean Difference IV, Random, 95% CI
**3.1.1 Jinshuibao capsule**								
Lin2024	0	0	0	0	0	0		Not estimable
Ling 2021	0	0	0	0	0	0		Not estimable
Tang 2024	0	0	0	0	0	0		Not estimable
Wang2023	6.72	1.48	30	7.39	1.46	30	6.8%	-0.45 [-0.96, 0.06]
Ye 2012	0	0	0	0	0	0		Not estimable
Zhang2022	6.47	1.53	44	8.12	2.21	42	6.8%	-0.86 [-1.31, -0.42]
**Subtotal (95% CI)**			**74**			**72**	**13.6%**	**-0.68 [-1.08, -0.27]**
Heterogeneity: Tau² = 0.03; Chi² = 1.43, df = 1 (P = 0.23); I² = 30%								
Test for overall effect: Z = 3.29 (P = 0.0010)								
**3.1.2 Bailing capsule**								
Chen 2017	3.15	1.06	25	4.85	1.62	25	6.7%	-1.22 [-1.83, -0.61]
Cui 2020	8.92	2.98	66	4.12	1.01	34	6.8%	1.91 [1.41, 2.40]
Gao 2019	4.56	0.87	41	6.73	1.08	41	6.7%	-2.19 [-2.74, -1.64]
Huang 2018	3.56	0.3	50	6.62	0.29	50	6.0%	-10.29 [-11.80, -8.78]
Li2023	0	0	0	0	0	0		Not estimable
Liu 2014	6.2	2.3	28	6.8	2.1	28	6.8%	-0.27 [-0.80, 0.26]
Sun 2011	2.4	1.4	69	9.3	3.5	33	6.7%	-2.99 [-3.58, -2.40]
Sun 2012	3.1	1.5	53	9.8	2.5	49	6.7%	-3.26 [-3.86, -2.66]
Wangb 2019	5.1	1.2	28	9.7	1.4	28	6.6%	-3.48 [-4.33, -2.63]
Wu 2012	7.5	1.99	42	5.01	1.37	21	6.7%	1.36 [0.78, 1.94]
Zeng2023	0	0	0	0	0	0		Not estimable
Zhanga 2020	6.3	0.83	43	9.18	1.21	43	6.7%	-2.75 [-3.35, -2.15]
Zhao2022	1.28	0.57	40	2.79	0.88	40	6.8%	-2.02 [-2.56, -1.47]
Zhu 2019	6.42	1.34	45	9.92	1.63	45	6.8%	-2.33 [-2.87, -1.79]
Zhu 2020	2.06	0.34	39	5.19	0.71	39	6.5%	-5.57 [-6.57, -4.57]
**Subtotal (95% CI)**			**569**			**476**	**86.4%**	**-2.48 [-3.70, -1.26]**
Heterogeneity: Tau² = 4.87; Chi² = 605.87, df = 12 (P < 0.00001); I² = 98%								
Test for overall effect: Z = 3.99 (P < 0.0001)								
**Total (95% CI)**			**643**			**548**	**100.0%**	**-2.22 [-3.24, -1.20]**
Heterogeneity: Tau² = 3.97; Chi² = 628.64, df = 14 (P < 0.00001); I² = 98%								
Test for overall effect: Z = 4.25 (P < 0.0001)								
Test for subgroup differences: Chi² = 7.57, df = 1 (P = 0.006), I² = 86.8%								

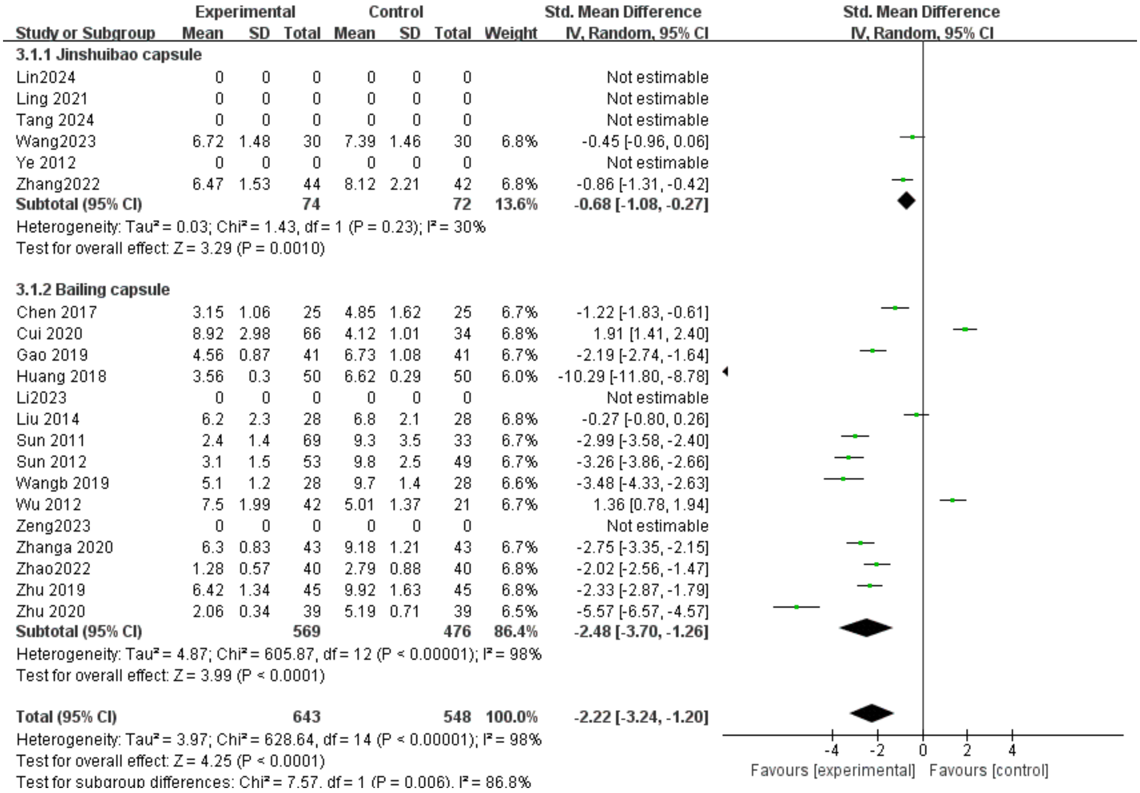

Fig.S3A

Study or Subgroup	Experimental Mean	Experimental SD	Experimental Total	Control Mean	Control SD	Control Total	Weight	Std. Mean Difference IV, Random, 95% CI
**3.2.1 Jinshuibao capsule**								
Lin2024	0	0	0	0	0	0		Not estimable
Ling 2021	0	0	0	0	0	0		Not estimable
Tang 2024	0	0	0	0	0	0		Not estimable
Wang2023	0	0	0	0	0	0		Not estimable
Ye 2012	0	0	0	0	0	0		Not estimable
Zhang2022	41.19	3.85	44	35.49	4.67	42	5.0%	1.32 [0.85, 1.79]
**Subtotal (95% CI)**			**44**			**42**	**5.0%**	**1.32 [0.85, 1.79]**
Heterogeneity: Not applicable								
Test for overall effect: Z = 5.53 (P < 0.00001)								
**3.2.2 Bailing capsule**								
Cui 2020	35.42	4.68	66	42.41	5.18	34	5.0%	-1.43 [-1.89, -0.97]
Gao 2019	43.25	1.45	41	40.76	1.8	41	5.0%	1.51 [1.02, 2.00]
Huang 2018	39.65	6.56	50	35.62	6.23	50	5.1%	0.63 [0.22, 1.03]
Li2023	44.82	7.28	65	41.19	5.31	65	5.2%	0.57 [0.22, 0.92]
Liu 2014	41.9	2.7	28	41.2	2.3	28	4.9%	0.28 [-0.25, 0.80]
Sun 2011	36.9	10.8	69	29.8	7.5	33	5.1%	0.71 [0.29, 1.14]
Sun 2012	36	10	53	30	8	49	5.1%	0.65 [0.26, 1.05]
Tian 2014	29.51	4.97	30	30.16	5.81	28	4.9%	-0.12 [-0.63, 0.40]
Tian 2021	35.31	4.41	30	33.11	5.27	30	4.9%	0.45 [-0.07, 0.96]
Wang 2016	42.1	3.2	30	41.5	3.4	30	4.9%	0.18 [-0.33, 0.69]
Wanga 2019	42.36	2.68	75	38.46	3.27	75	5.2%	1.30 [0.94, 1.65]
Wu 2012	32.56	11.38	42	33.12	9.66	21	4.9%	-0.05 [-0.57, 0.47]
Yang 2019	39.12	5.98	36	34.59	6.02	36	5.0%	0.75 [0.27, 1.23]
Ying 2018	42.31	1.78	47	39.46	1.51	47	5.0%	1.71 [1.24, 2.19]
Zeng2023	36.29	1.25	54	31.14	1.42	54	4.7%	3.82 [3.18, 4.46]
Zhangb 2020	38.43	3.08	47	31.61	2.17	47	4.9%	2.54 [1.99, 3.09]
Zhao2022	41.17	5.46	40	40.03	5.21	40	5.0%	0.21 [-0.23, 0.65]
Zhu 2019	42.19	4.83	45	39.73	4.91	45	5.1%	0.50 [0.08, 0.92]
Zhu 2020	45.52	4.67	39	41.68	4.73	39	5.0%	0.81 [0.35, 1.27]
**Subtotal (95% CI)**			**887**			**792**	**95.0%**	**0.78 [0.36, 1.20]**
Heterogeneity: Tau² = 0.81; Chi² = 286.60, df = 18 (P < 0.00001); I² = 94%								
Test for overall effect: Z = 3.65 (P = 0.0003)								
**Total (95% CI)**			**931**			**834**	**100.0%**	**0.81 [0.41, 1.21]**
Heterogeneity: Tau² = 0.78; Chi² = 292.79, df = 19 (P < 0.00001); I² = 94%								
Test for overall effect: Z = 3.94 (P < 0.0001)								
Test for subgroup differences: Chi² = 2.86, df = 1 (P = 0.09), I² = 65.0%								

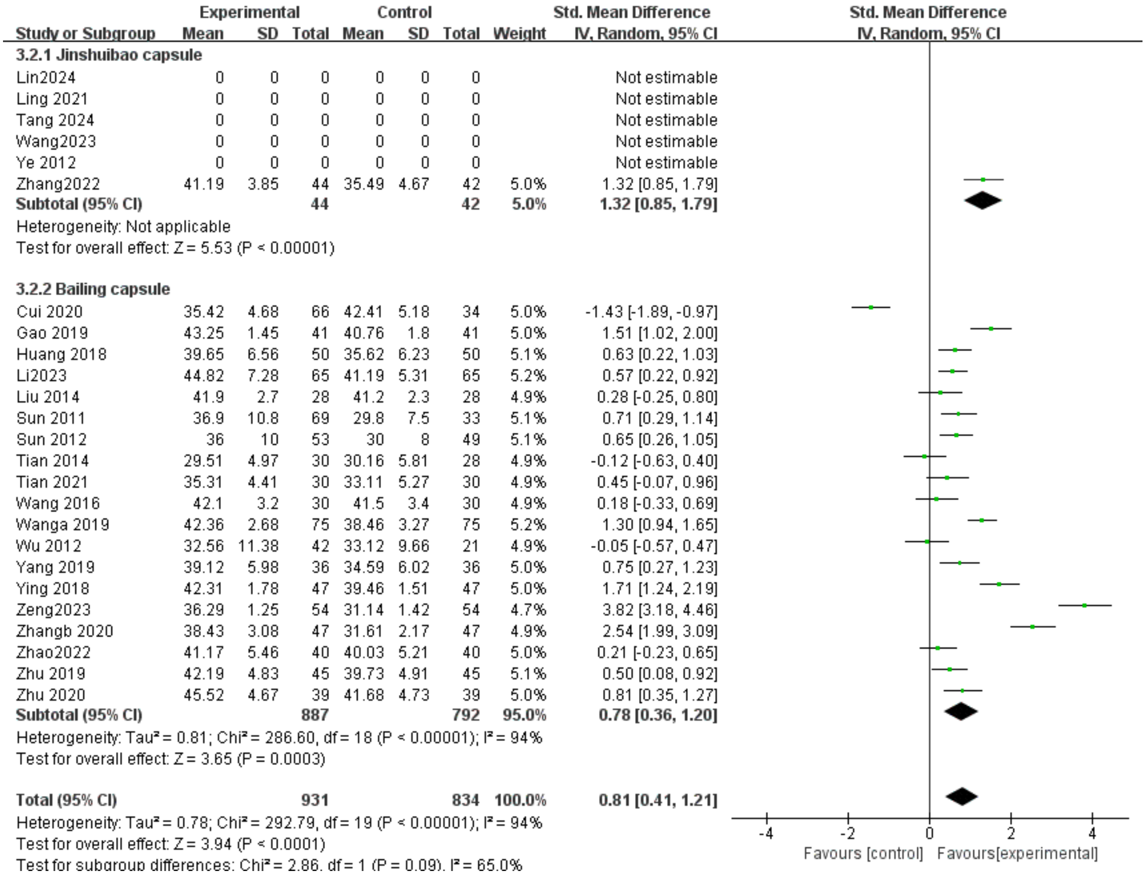

Fig.S3B

Study or Subgroup	Experimental Mean	Experimental SD	Experimental Total	Control Mean	Control SD	Control Total	Weight	Std. Mean Difference IV, Random, 95% CI
**3.3.1 Jinshuibao capsule**								
Lin2024	115.53	12.53	40	108.64	11.44	40	5.4%	0.57 [0.12, 1.02]
Ling 2021	102.35	7.83	41	92.67	7.65	41	5.3%	1.24 [0.76, 1.71]
Tang 2024	0	0	0	0	0	0		Not estimable
Wang2023	89.07	9.26	30	83.36	12.3	30	5.2%	0.52 [0.00, 1.03]
Ye 2012	104.69	12.46	34	106.72	13.09	34	5.3%	-0.16 [-0.63, 0.32]
Zhang2022	0	0	0	0	0	0		Not estimable
**Subtotal (95% CI)**			**145**			**145**	**21.1%**	**0.54 [-0.02, 1.10]**
Heterogeneity: Tau² = 0.27; Chi² = 16.59, df = 3 (P = 0.0009); I² = 82%								
Test for overall effect: Z = 1.89 (P = 0.06)								
**3.3.2 Bailing capsule**								
Gao 2019	108.42	9.36	41	94.65	4.78	41	5.2%	1.84 [1.32, 2.36]
He 2021	106.5	9.44	38	95.69	8.31	38	5.3%	1.20 [0.71, 1.69]
Li2023	92.83	8.24	65	86.1	9.75	65	5.5%	0.74 [0.39, 1.10]
Liu 2014	102.4	8.3	28	98.7	9.4	28	5.2%	0.41 [-0.12, 0.94]
Ren 2021	147.91	6.45	55	125.57	6.41	55	5.0%	3.45 [2.85, 4.05]
Sun 2011	123.1	19	69	97.4	9.2	33	5.3%	1.54 [1.08, 2.01]
Sun 2012	119	12	53	96	9	49	5.3%	2.14 [1.65, 2.63]
Tian 2014	89.4	11.61	30	88.16	12.64	28	5.2%	0.10 [-0.41, 0.62]
Tian 2021	89.2	11.56	30	77.18	12.24	30	5.2%	1.00 [0.46, 1.54]
Wang 2016	102.6	8.5	30	99.8	9.6	30	5.2%	0.30 [-0.20, 0.81]
Yang 2019	93.74	11.28	36	84.01	9.83	36	5.3%	0.91 [0.42, 1.40]
Ying 2018	98.42	3.25	47	94.25	2.72	47	5.3%	1.38 [0.93, 1.83]
Zeng2023	109.38	6.24	54	95.21	6.28	54	5.3%	2.25 [1.76, 2.73]
Zhao2022	0	0	0	0	0	0		Not estimable
Zhu 2019	103.64	11.09	45	94.86	10.32	45	5.4%	0.81 [0.38, 1.24]
Zhu 2020	108.67	8.51	39	97.85	8.13	39	5.3%	1.29 [0.80, 1.78]
**Subtotal (95% CI)**			**660**			**618**	**78.9%**	**1.28 [0.88, 1.69]**
Heterogeneity: Tau² = 0.56; Chi² = 144.39, df = 14 (P < 0.00001); I² = 90%								
Test for overall effect: Z = 6.29 (P < 0.00001)								
**Total (95% CI)**			**805**			**763**	**100.0%**	**1.13 [0.77, 1.48]**
Heterogeneity: Tau² = 0.56; Chi² = 185.82, df = 18 (P < 0.00001); I² = 90%								
Test for overall effect: Z = 6.23 (P < 0.00001)								
Test for subgroup differences: Chi² = 4.45, df = 1 (P = 0.03), I² = 77.5%								

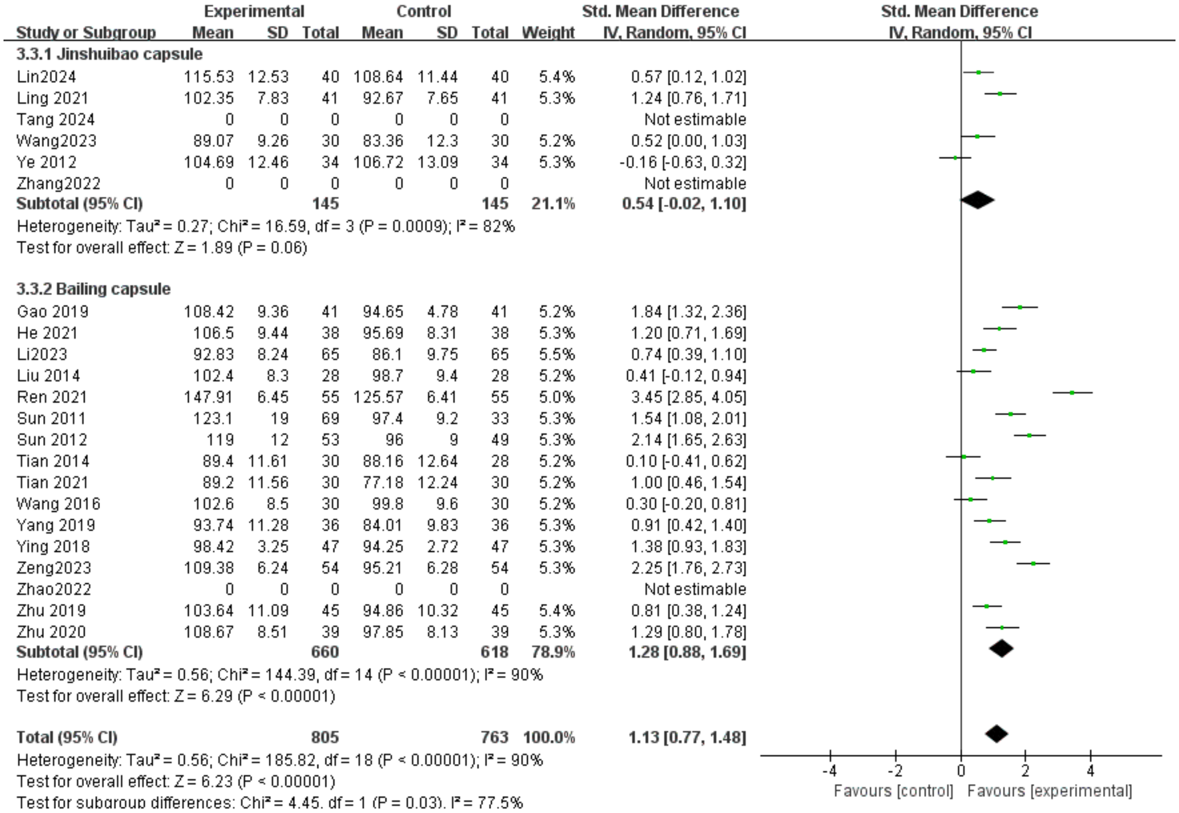

Fig.S3C

Study or Subgroup	Experimental Mean	Experimental SD	Experimental Total	Control Mean	Control SD	Control Total	Weight	Std. Mean Difference IV, Random, 95% CI
**3.4.1 Jinshuibao capsule**								
Lin2024	315.44	25.66	40	362.18	21.35	40	4.6%	-1.96 [-2.50, -1.42]
Ling 2021	248.25	22.34	41	301.64	30.47	41	4.6%	-1.98 [-2.51, -1.45]
Tang 2024	385.73	102.62	63	423.64	103.55	63	4.7%	-0.37 [-0.72, -0.01]
Wang2023	0	0	0	0	0	0		Not estimable
Ye 2012	575.69	29.97	34	581.27	30.21	34	4.6%	-0.18 [-0.66, 0.29]
Zhang2022	157.95	12.47	44	188.76	19.68	42	4.6%	-1.86 [-2.37, -1.35]
**Subtotal (95% CI)**			**222**			**220**	**23.1%**	**-1.26 [-2.07, -0.44]**
Heterogeneity: Tau² = 0.81; Chi² = 59.70, df = 4 (P < 0.00001); I² = 93%								
Test for overall effect: Z = 3.02 (P = 0.003)								
**3.4.2 Bailing capsule**								
Gao 2019	175.52	16.85	41	234.51	23.27	41	4.5%	-2.88 [-3.50, -2.25]
Li 2021	604.3	22.42	25	697.88	22.25	25	3.9%	-4.12 [-5.13, -3.12]
Li2023	324.75	84.6	65	385.4	93.47	65	4.7%	-0.68 [-1.03, -0.32]
Liu 2014	916.8	102.7	28	923.3	107.4	28	4.6%	-0.06 [-0.58, 0.46]
Ren 2021	287.89	21.14	55	325.41	21.12	55	4.7%	-1.76 [-2.21, -1.32]
Tian 2014	945.76	311.54	30	988.94	321.58	28	4.6%	-0.13 [-0.65, 0.38]
Tian 2021	341.4	108.41	30	660.71	185.27	30	4.5%	-2.08 [-2.71, -1.44]
Wang 2018	765	68	40	762	54	40	4.7%	0.05 [-0.39, 0.49]
Wanga 2019	832.52	84.25	75	902.35	96.57	75	4.7%	-0.77 [-1.10, -0.43]
Wangb 2019	697.5	53.1	28	849.7	60.3	28	4.3%	-2.64 [-3.37, -1.91]
Wu 2012	783.9	77.06	42	694.54	52.33	21	4.5%	1.26 [0.69, 1.83]
Xie 2019	314.5	18.5	50	357.4	20.1	50	4.6%	-2.20 [-2.70, -1.70]
Yao 2016	546.58	109.09	17	541.67	114.78	18	4.4%	0.04 [-0.62, 0.71]
Ying 2018	845.29	7.56	47	885.13	8.98	47	4.2%	-4.76 [-5.56, -3.96]
Zeng2023	831.24	61.39	54	892.53	55.1	54	4.7%	-1.04 [-1.45, -0.64]
Zhanga 2020	453.77	48.76	43	510.33	55.45	43	4.6%	-1.07 [-1.53, -0.62]
Zhao2022	451.46	51.24	40	497.65	59.38	40	4.6%	-0.82 [-1.28, -0.37]
**Subtotal (95% CI)**			**710**			**688**	**76.9%**	**-1.35 [-1.92, -0.79]**
Heterogeneity: Tau² = 1.32; Chi² = 340.12, df = 16 (P < 0.00001); I² = 95%								
Test for overall effect: Z = 4.71 (P < 0.00001)								
**Total (95% CI)**			**932**			**908**	**100.0%**	**-1.33 [-1.79, -0.87]**
Heterogeneity: Tau² = 1.13; Chi² = 399.90, df = 21 (P < 0.00001); I² = 95%								
Test for overall effect: Z = 5.66 (P < 0.00001)								
Test for subgroup differences: Chi² = 0.03, df = 1 (P = 0.85), I² = 0%								

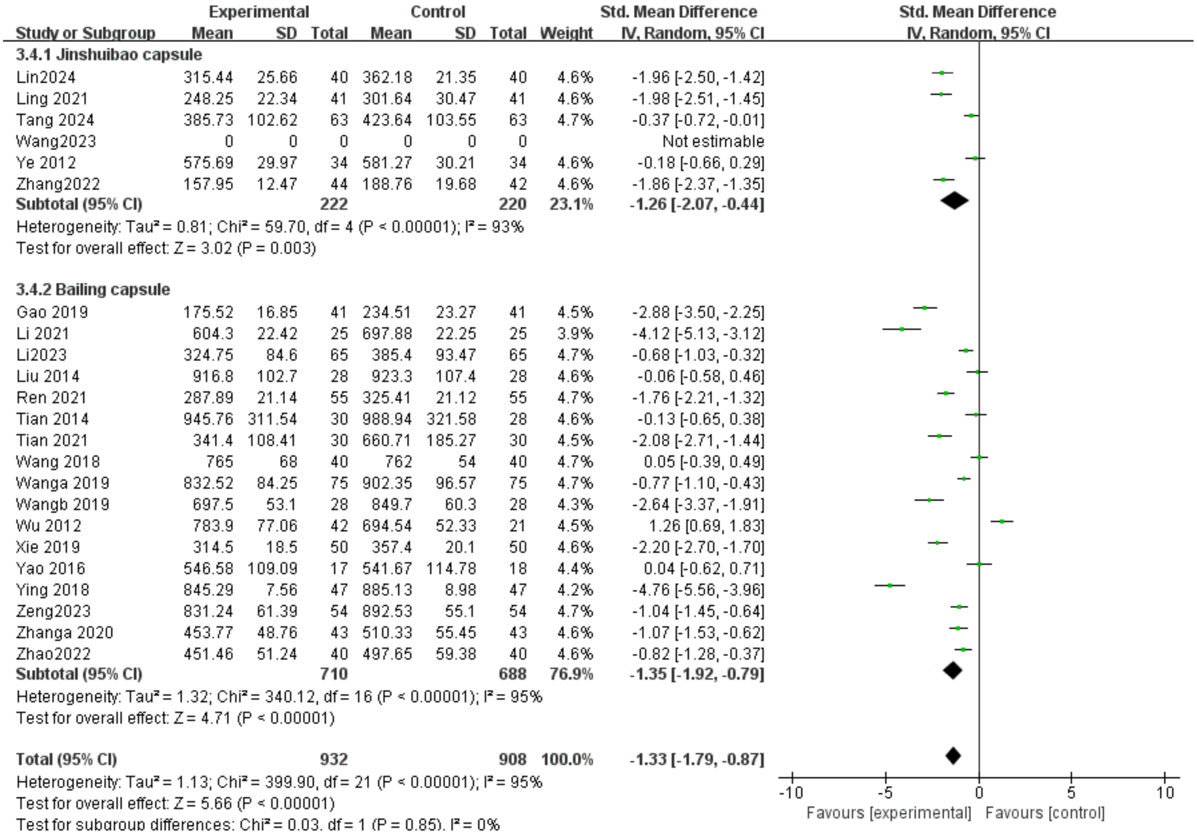

Fig.S3D

Study or Subgroup	Experimental Mean	Experimental SD	Experimental Total	Control Mean	Control SD	Control Total	Weight	Std. Mean Difference IV, Random, 95% CI
**3.5.1 Jinshuibao capsule**								
Lin2024	14.27	2.37	40	16.15	1.18	40	5.3%	-0.99 [-1.46, -0.53]
Ling 2021	11.33	2.05	41	15.78	2.34	41	5.3%	-2.00 [-2.54, -1.47]
Tang 2024	13.82	2.19	63	17.94	2.21	63	5.4%	-1.86 [-2.28, -1.44]
Wang2023	0	0	0	0	0	0		Not estimable
Ye 2012	21.63	3.19	34	22.57	3.91	34	5.3%	-0.26 [-0.74, 0.22]
Zhang2022	15.54	2.25	44	19.69	2.63	42	5.3%	-1.68 [-2.18, -1.19]
**Subtotal (95% CI)**			**222**			**220**	**26.7%**	**-1.36 [-1.99, -0.73]**
Heterogeneity: Tau² = 0.46; Chi² = 35.41, df = 4 (P < 0.00001); I² = 89%								
Test for overall effect: Z = 4.20 (P < 0.0001)								
**3.5.2 Bailing capsule**								
Gao 2019	13.26	2.87	41	18.98	2.33	41	5.3%	-2.17 [-2.72, -1.62]
Li 2021	21.5	3.74	25	38.74	4.71	25	4.7%	-3.99 [-4.98, -3.00]
Li2023	12.15	2.96	65	19.75	1.26	65	5.3%	-3.32 [-3.86, -2.79]
Liu 2014	26.3	5.2	28	27.4	5.1	28	5.3%	-0.21 [-0.74, 0.31]
Ren 2021	12.84	1.69	55	18.71	1.62	55	5.2%	-3.52 [-4.12, -2.92]
Tian 2014	18.21	7.82	30	17.13	6.43	28	5.3%	0.15 [-0.37, 0.66]
Tian 2021	14.37	4.6	30	25.45	7.24	30	5.2%	-1.80 [-2.41, -1.20]
Wang 2018	26.7	6.7	40	27.6	4.3	40	5.4%	-0.16 [-0.60, 0.28]
Wanga 2019	21.31	3.28	75	25.14	4.37	75	5.5%	-0.99 [-1.33, -0.65]
Wangb 2019	16.1	3.8	28	28.9	7.3	28	5.1%	-2.17 [-2.84, -1.50]
Wu 2012	24.72	7.41	42	15.77	3.81	21	5.2%	1.37 [0.79, 1.95]
Xie 2019	13.7	2.3	50	17.6	2.5	50	5.4%	-1.61 [-2.06, -1.16]
Ying 2018	24.21	0.87	47	26.65	1.04	47	5.3%	-2.52 [-3.07, -1.98]
Zhanga 2020	12.18	1.96	43	15.01	2.46	43	5.3%	-1.26 [-1.73, -0.80]
**Subtotal (95% CI)**			**599**			**576**	**73.3%**	**-1.57 [-2.27, -0.86]**
Heterogeneity: Tau² = 1.73; Chi² = 332.74, df = 13 (P < 0.00001); I² = 96%								
Test for overall effect: Z = 4.35 (P < 0.0001)								
**Total (95% CI)**			**821**			**796**	**100.0%**	**-1.51 [-2.03, -0.98]**
Heterogeneity: Tau² = 1.30; Chi² = 368.19, df = 18 (P < 0.00001); I² = 95%								
Test for overall effect: Z = 5.60 (P < 0.00001)								
Test for subgroup differences: Chi² = 0.18, df = 1 (P = 0.67), I² = 0%								

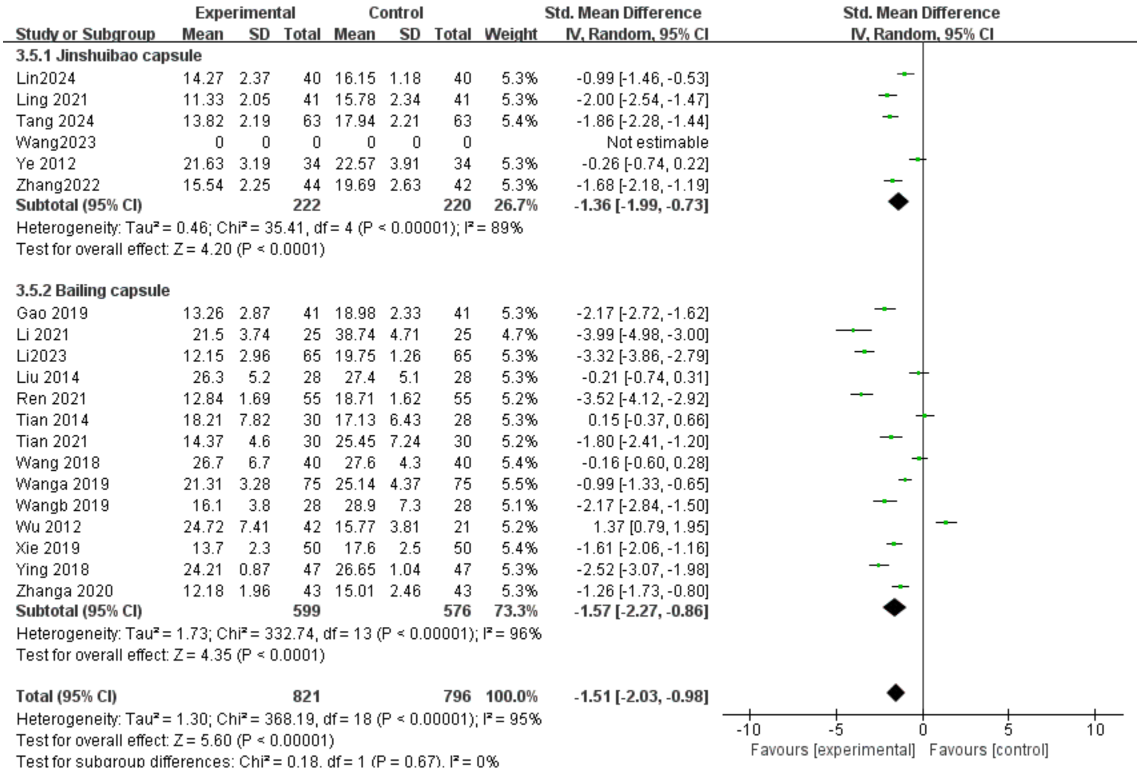

Fig.S3E

	Experimental			Control				Std. Mean Difference
**Study or Subgroup**	**Mean**	**SD**	**Total**	**Mean**	**SD**	**Total**	**Weight**	**IV, Random, 95% CI**
**4.1.1 ≤12weeks**								
Chen 2017	3.15	1.06	25	4.85	1.62	25	6.7%	-1.22 [-1.83, -0.61]
Cui 2020	8.92	2.98	66	4.12	1.01	34	6.8%	1.91 [1.41, 2.40]
Gao 2019	4.56	0.87	41	6.73	1.08	41	6.7%	-2.19 [-2.74, -1.64]
Huang 2018	3.56	0.3	50	6.62	0.29	50	6.0%	-10.29 [-11.80, -8.78]
Li2023	0	0	0	0	0	0		Not estimable
Lin2024	0	0	0	0	0	0		Not estimable
Tang 2024	0	0	0	0	0	0		Not estimable
Wu 2012	7.5	1.99	42	5.01	1.37	21	6.7%	1.36 [0.78, 1.94]
Zeng2023	0	0	0	0	0	0		Not estimable
Zhang2022	6.47	1.53	44	8.12	2.21	42	6.8%	-0.86 [-1.31, -0.42]
Zhanga 2020	6.3	0.83	43	9.18	1.21	43	6.7%	-2.75 [-3.35, -2.15]
Zhao2022	1.28	0.57	40	2.79	0.88	40	6.8%	-2.02 [-2.56, -1.47]
Zhu 2019	6.42	1.34	45	9.92	1.63	45	6.8%	-2.33 [-2.87, -1.79]
**Subtotal (95% CI)**			**396**			**341**	**60.0%**	**-1.95 [-3.35, -0.55]**
Heterogeneity: Tau² = 4.47; Chi² = 431.06, df = 8 (P < 0.00001); I² = 98%								
Test for overall effect: Z = 2.73 (P = 0.006)								
**4.1.2 >12weeks**								
Liu 2014	6.2	2.3	28	6.8	2.1	28	6.8%	-0.27 [-0.80, 0.26]
Sun 2011	2.4	1.4	69	9.3	3.5	33	6.7%	-2.99 [-3.58, -2.40]
Sun 2012	3.1	1.5	53	9.8	2.5	49	6.7%	-3.26 [-3.86, -2.66]
Wang2023	6.72	1.48	30	7.39	1.46	30	6.8%	-0.45 [-0.96, 0.06]
Wangb 2019	5.1	1.2	28	9.7	1.4	28	6.6%	-3.48 [-4.33, -2.63]
Zhu 2020	2.06	0.34	39	5.19	0.71	39	6.5%	-5.57 [-6.57, -4.57]
**Subtotal (95% CI)**			**247**			**207**	**40.0%**	**-2.64 [-4.13, -1.15]**
Heterogeneity: Tau² = 3.34; Chi² = 164.86, df = 5 (P < 0.00001); I² = 97%								
Test for overall effect: Z = 3.47 (P = 0.0005)								
**Total (95% CI)**			**643**			**548**	**100.0%**	**-2.22 [-3.24, -1.20]**
Heterogeneity: Tau² = 3.97; Chi² = 628.64, df = 14 (P < 0.00001); I² = 98%								
Test for overall effect: Z = 4.25 (P < 0.0001)								
Test for subgroup differences: Chi² = 0.43, df = 1 (P = 0.51), I² = 0%								

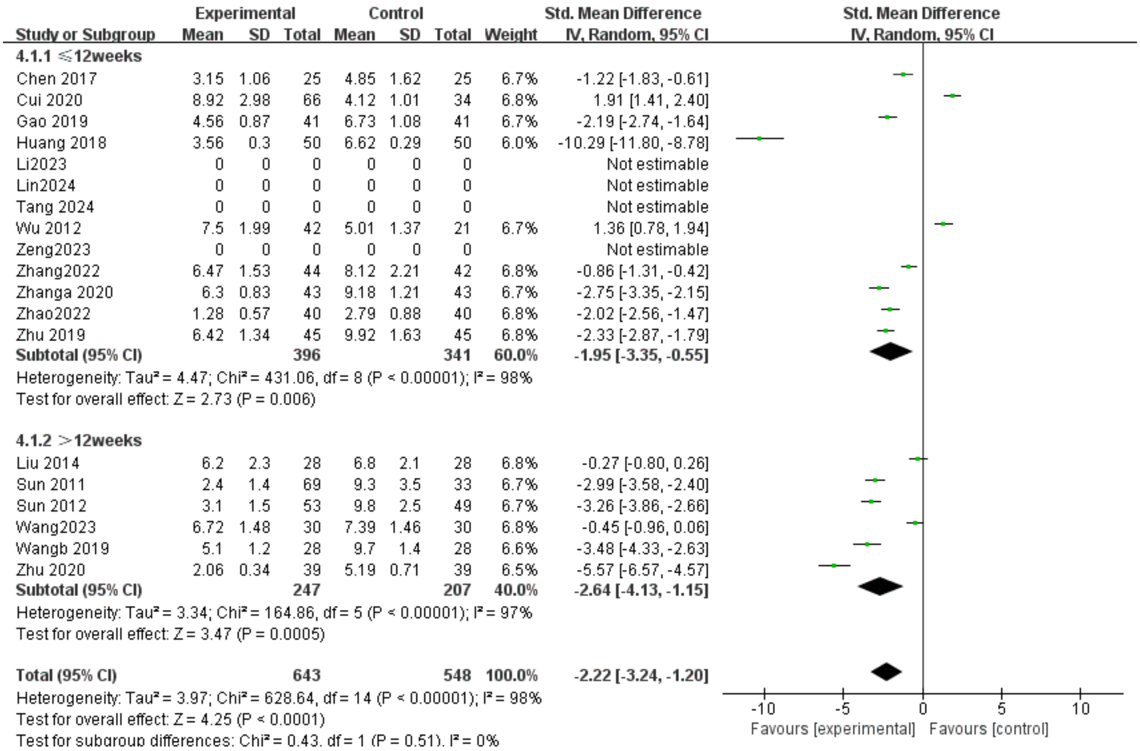

Fig.S4A

Study or Subgroup	Experimental Mean	Experimental SD	Experimental Total	Control Mean	Control SD	Control Total	Weight	Std. Mean Difference IV, Random, 95% CI
**4.2.1 ≤12weeks**								
Cui 2020	35.42	4.68	66	42.41	5.18	34	5.3%	-1.43 [-1.89, -0.97]
Gao 2019	43.25	1.45	41	40.76	1.8	41	5.2%	1.51 [1.02, 2.00]
Huang 2018	39.65	6.56	50	35.62	6.23	50	5.4%	0.63 [0.22, 1.03]
Li2023	44.82	7.28	65	41.19	5.31	65	5.4%	0.57 [0.22, 0.92]
Lin2024	0	0	0	0	0	0		Not estimable
Tang 2024	0	0	0	0	0	0		Not estimable
Tian 2014	29.51	4.97	30	30.16	5.81	28	5.2%	-0.12 [-0.63, 0.40]
Tian 2021	35.31	4.41	30	33.11	5.27	30	5.2%	0.45 [-0.07, 0.96]
Wanga 2019	42.36	2.68	75	38.46	3.27	75	5.4%	1.30 [0.94, 1.65]
Wu 2012	32.56	11.38	42	33.12	9.66	21	5.2%	-0.05 [-0.57, 0.47]
Zeng2023	36.29	1.25	54	31.14	1.42	54	4.9%	3.82 [3.18, 4.46]
Zhang2022	41.19	3.85	44	35.49	4.67	42	5.3%	1.32 [0.85, 1.79]
Zhao2022	41.17	5.46	40	40.03	5.21	40	5.3%	0.21 [-0.23, 0.65]
Zhu 2019	42.19	4.83	45	39.73	4.91	45	5.3%	0.50 [0.08, 0.92]
**Subtotal (95% CI)**			**582**			**525**	**63.2%**	**0.72 [0.13, 1.30]**
Heterogeneity: Tau² = 1.02; Chi² = 225.22, df = 11 (P < 0.00001); I² = 95%								
Test for overall effect: Z = 2.39 (P = 0.02)								
**4.2.2 >12weeks**								
Liu 2014	41.9	2.7	28	41.2	2.3	28	5.2%	0.28 [-0.25, 0.80]
Sun 2011	36.9	10.8	69	29.8	7.5	33	5.3%	0.71 [0.29, 1.14]
Sun 2012	36	10	53	30	8	49	5.4%	0.65 [0.26, 1.05]
Wang 2016	42.1	3.2	30	41.5	3.4	30	5.2%	0.18 [-0.33, 0.69]
Wang2023	0	0	0	0	0	0		Not estimable
Yang 2019	39.12	5.98	36	34.59	6.02	36	5.2%	0.75 [0.27, 1.23]
Ying 2018	42.31	1.78	47	39.46	1.51	47	5.3%	1.71 [1.24, 2.19]
Zhu 2020	45.52	4.67	39	41.68	4.73	39	5.3%	0.81 [0.35, 1.27]
**Subtotal (95% CI)**			**302**			**262**	**36.8%**	**0.73 [0.38, 1.08]**
Heterogeneity: Tau² = 0.17; Chi² = 24.04, df = 6 (P = 0.0005); I² = 75%								
Test for overall effect: Z = 4.08 (P < 0.0001)								
**Total (95% CI)**			**884**			**787**	**100.0%**	**0.72 [0.33, 1.11]**
Heterogeneity: Tau² = 0.69; Chi² = 250.11, df = 18 (P < 0.00001); I² = 93%								
Test for overall effect: Z = 3.62 (P = 0.0003)								
Test for subgroup differences: Chi² = 0.00, df = 1 (P = 0.96), I² = 0%								

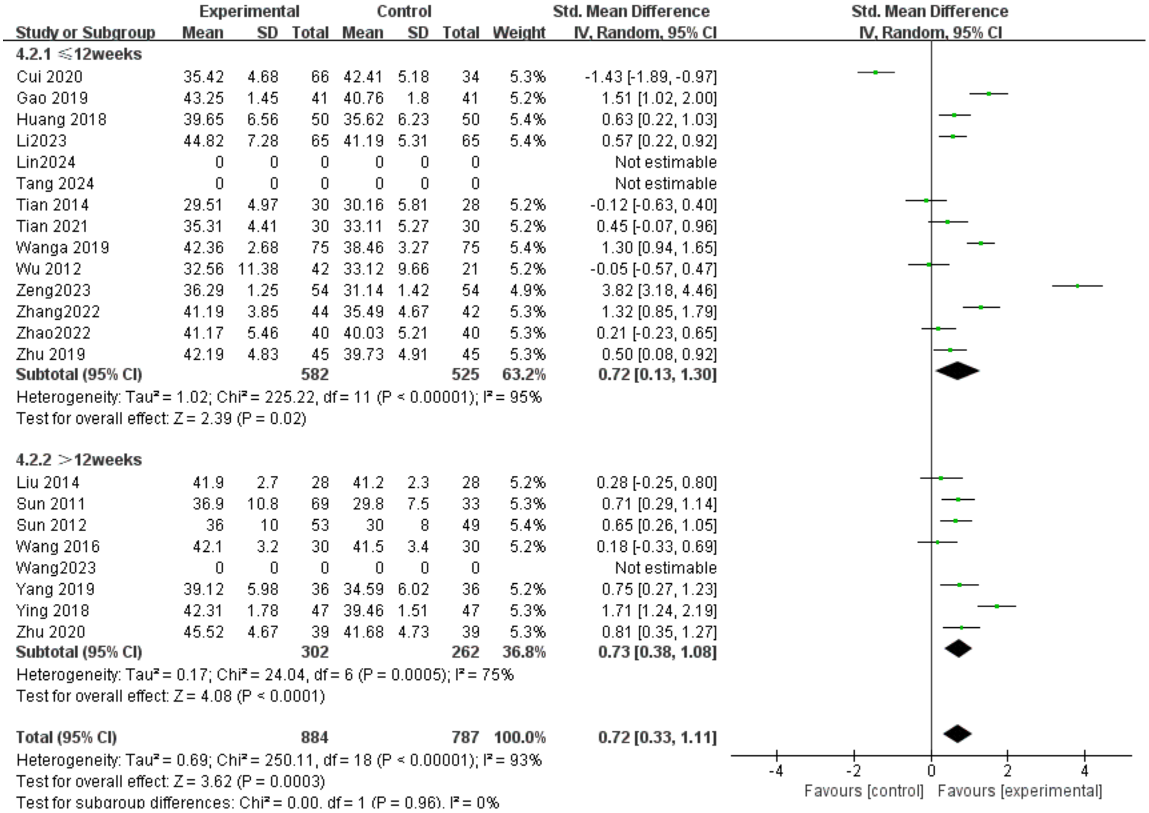

Fig.S4B

Study or Subgroup	Experimental Mean	Experimental SD	Experimental Total	Control Mean	Control SD	Control Total	Weight	Std. Mean Difference IV, Random, 95% CI
**4.3.1 ≤12weeks**								
Gao 2019	108.42	9.36	41	94.65	4.78	41	5.2%	1.84 [1.32, 2.36]
He 2021	106.5	9.44	38	95.69	8.31	38	5.3%	1.20 [0.71, 1.69]
Li2023	92.83	8.24	65	86.1	9.75	65	5.5%	0.74 [0.39, 1.10]
Lin2024	115.53	12.53	40	108.64	11.44	40	5.4%	0.57 [0.12, 1.02]
Ling 2021	102.35	7.83	41	92.67	7.65	41	5.3%	1.24 [0.76, 1.71]
Ren 2021	147.91	6.45	55	125.57	6.41	55	5.0%	3.45 [2.85, 4.05]
Tang 2024	0	0	0	0	0	0		Not estimable
Tian 2014	89.4	11.61	30	88.16	12.64	28	5.2%	0.10 [-0.41, 0.62]
Tian 2021	89.2	11.56	30	77.18	12.24	30	5.2%	1.00 [0.46, 1.54]
Ye 2012	104.69	12.46	34	106.72	13.09	34	5.3%	-0.16 [-0.63, 0.32]
Zeng2023	109.38	6.24	54	95.21	6.28	54	5.3%	2.25 [1.76, 2.73]
Zhang2022	0	0	0	0	0	0		Not estimable
Zhao2022	0	0	0	0	0	0		Not estimable
Zhu 2019	103.64	11.09	45	94.86	10.32	45	5.4%	0.81 [0.38, 1.24]
**Subtotal (95% CI)**			**473**			**471**	**58.0%**	**1.18 [0.64, 1.71]**
Heterogeneity: Tau² = 0.77; Chi² = 141.61, df = 10 (P < 0.00001); I² = 93%								
Test for overall effect: Z = 4.27 (P < 0.0001)								
**4.3.2 ＞12weeks**								
Liu 2014	102.4	8.3	28	98.7	9.4	28	5.2%	0.41 [-0.12, 0.94]
Sun 2011	123.1	19	69	97.4	9.2	33	5.3%	1.54 [1.08, 2.01]
Sun 2012	119	12	53	96	9	49	5.3%	2.14 [1.65, 2.63]
Wang 2016	102.6	8.5	30	99.8	9.6	30	5.2%	0.30 [-0.20, 0.81]
Wang2023	89.07	9.26	30	83.36	12.3	30	5.2%	0.52 [0.00, 1.03]
Yang 2019	93.74	11.28	36	84.01	9.83	36	5.3%	0.91 [0.42, 1.40]
Ying 2018	98.42	3.25	47	94.25	2.72	47	5.3%	1.38 [0.93, 1.83]
Zhu 2020	108.67	8.51	39	97.85	8.13	39	5.3%	1.29 [0.80, 1.78]
**Subtotal (95% CI)**			**332**			**292**	**42.0%**	**1.07 [0.63, 1.50]**
Heterogeneity: Tau² = 0.33; Chi² = 44.15, df = 7 (P < 0.00001); I² = 84%								
Test for overall effect: Z = 4.80 (P < 0.00001)								
**Total (95% CI)**			**805**			**763**	**100.0%**	**1.13 [0.77, 1.48]**
Heterogeneity: Tau² = 0.56; Chi² = 185.82, df = 18 (P < 0.00001); I² = 90%								
Test for overall effect: Z = 6.23 (P < 0.00001)								
Test for subgroup differences: Chi² = 0.09, df = 1 (P = 0.76), I² = 0%								

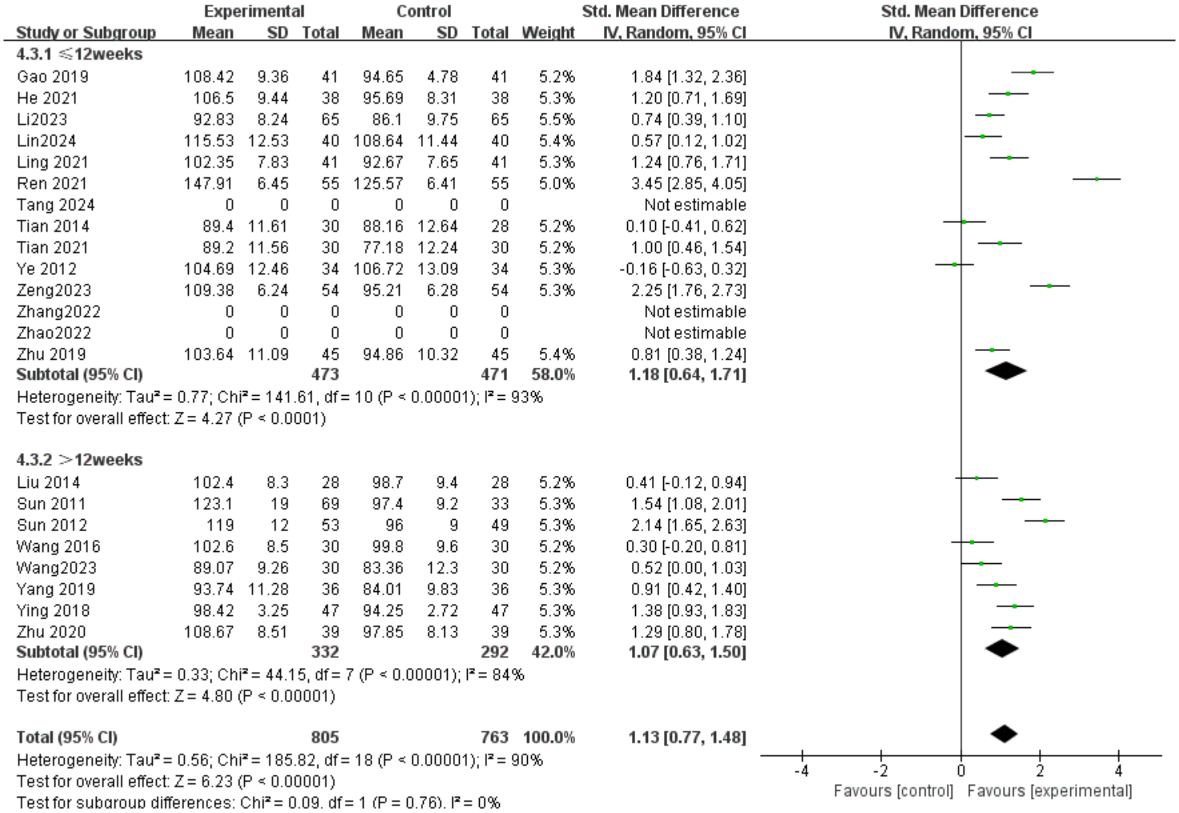

Fig.S4C

Study or Subgroup	Experimental Mean	Experimental SD	Experimental Total	Control Mean	Control SD	Control Total	Weight	Std. Mean Difference IV, Random, 95% CI
**4.4.1 ≤12weeks**								
Gao 2019	175.52	16.85	41	234.51	23.27	41	4.5%	-2.88 [-3.50, -2.25]
Li2023	324.75	84.6	65	385.4	93.47	65	4.7%	-0.68 [-1.03, -0.32]
Lin2024	315.44	25.66	40	362.18	21.35	40	4.6%	-1.96 [-2.50, -1.42]
Ling 2021	248.25	22.34	41	301.64	30.47	41	4.6%	-1.98 [-2.51, -1.45]
Ren 2021	287.89	21.14	55	325.41	21.12	55	4.7%	-1.76 [-2.21, -1.32]
Tang 2024	385.73	102.62	63	423.64	103.55	63	4.7%	-0.37 [-0.72, -0.01]
Tian 2014	945.76	311.54	30	988.94	321.58	28	4.6%	-0.13 [-0.65, 0.38]
Tian 2021	341.4	108.41	30	660.71	185.27	30	4.5%	-2.08 [-2.71, -1.44]
Wanga 2019	832.52	84.25	75	902.35	96.57	75	4.7%	-0.77 [-1.10, -0.43]
Wu 2012	783.9	77.06	42	694.54	52.33	21	4.5%	1.26 [0.69, 1.83]
Ye 2012	575.69	29.97	34	581.27	30.21	34	4.6%	-0.18 [-0.66, 0.29]
Zeng2023	831.24	61.39	54	892.53	55.1	54	4.7%	-1.04 [-1.45, -0.64]
Zhang2022	157.95	12.47	44	188.76	19.68	42	4.6%	-1.86 [-2.37, -1.35]
Zhanga 2020	453.77	48.76	43	510.33	55.45	43	4.6%	-1.07 [-1.53, -0.62]
Zhao2022	451.46	51.24	40	497.65	59.38	40	4.6%	-0.82 [-1.28, -0.37]
**Subtotal (95% CI)**			**697**			**672**	**69.2%**	**-1.08 [-1.52, -0.64]**
Heterogeneity: Tau² = 0.69; Chi² = 193.67, df = 14 (P < 0.00001); I² = 93%								
Test for overall effect: Z = 4.80 (P < 0.00001)								
**4.4.2 >12weeks**								
Li 2021	604.3	22.42	25	697.88	22.25	25	3.9%	-4.12 [-5.13, -3.12]
Liu 2014	916.8	102.7	28	923.3	107.4	28	4.6%	-0.06 [-0.58, 0.46]
Wang 2018	765	68	40	762	54	40	4.7%	0.05 [-0.39, 0.49]
Wang2023	0	0	0	0	0	0		Not estimable
Wangb 2019	697.5	53.1	28	849.7	60.3	28	4.3%	-2.64 [-3.37, -1.91]
Xie 2019	314.5	18.5	50	357.4	20.1	50	4.6%	-2.20 [-2.70, -1.70]
Yao 2016	546.58	109.09	17	541.67	114.78	18	4.4%	0.04 [-0.62, 0.71]
Ying 2018	845.29	7.56	47	885.13	8.98	47	4.2%	-4.76 [-5.56, -3.96]
**Subtotal (95% CI)**			**235**			**236**	**30.8%**	**-1.93 [-3.27, -0.59]**
Heterogeneity: Tau² = 3.16; Chi² = 200.35, df = 6 (P < 0.00001); I² = 97%								
Test for overall effect: Z = 2.82 (P = 0.005)								
**Total (95% CI)**			**932**			**908**	**100.0%**	**-1.33 [-1.79, -0.87]**
Heterogeneity: Tau² = 1.13; Chi² = 399.90, df = 21 (P < 0.00001); I² = 95%								
Test for overall effect: Z = 5.66 (P < 0.00001)								
Test for subgroup differences: Chi² = 1.39, df = 1 (P = 0.24), I² = 28.1%								

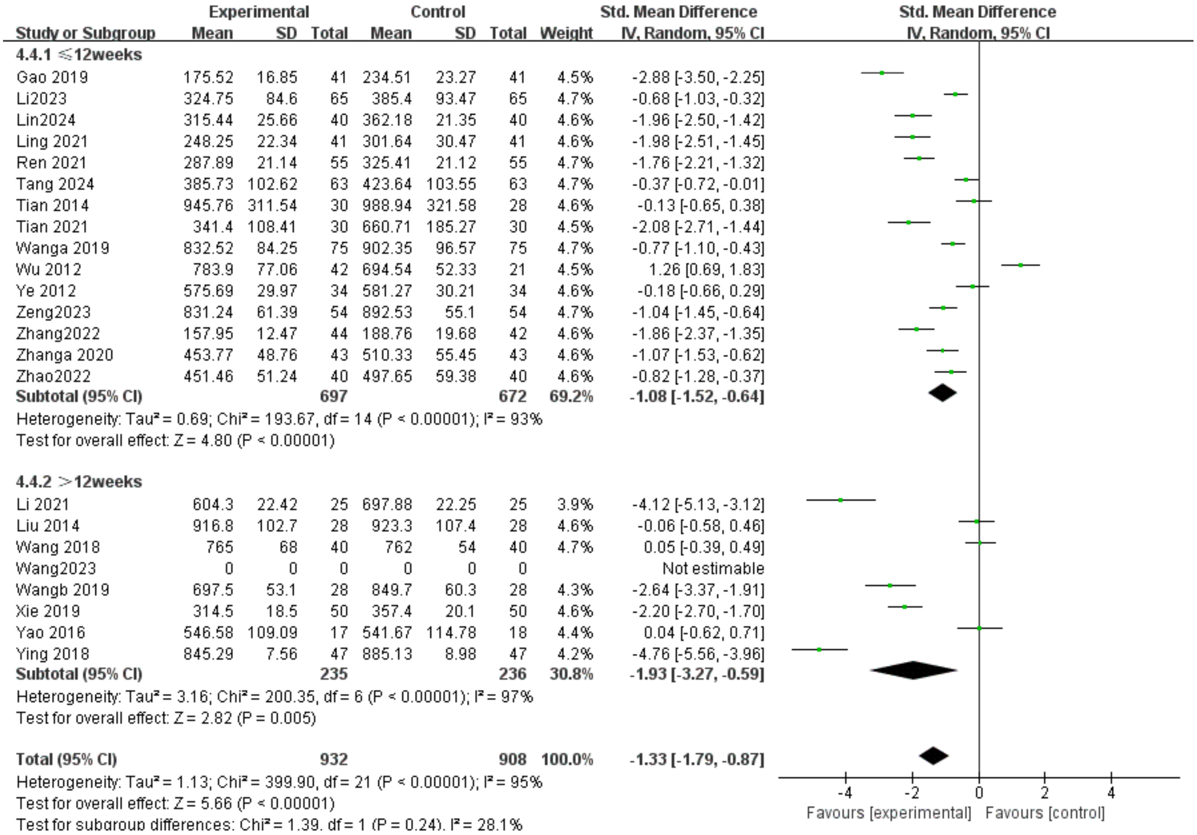

Fig.S4D

Study or Subgroup	Experimental Mean	Experimental SD	Experimental Total	Control Mean	Control SD	Control Total	Weight	Std. Mean Difference IV, Random, 95% CI
**4.5.1 ≤12weeks**								
Gao 2019	13.26	2.87	41	18.98	2.33	41	4.8%	-2.17 [-2.72, -1.62]
Li2023	12.15	2.96	65	19.75	1.26	65	4.8%	-3.32 [-3.86, -2.79]
Lin2024	14.27	2.37	40	16.15	1.18	40	4.8%	-0.99 [-1.46, -0.53]
Ling 2021	11.33	2.05	41	15.78	2.34	41	4.8%	-2.00 [-2.54, -1.47]
Ren 2021	12.84	1.69	55	18.71	1.62	55	4.7%	-3.52 [-4.12, -2.92]
Tang 2024	13.82	2.19	63	17.94	2.21	63	4.9%	-1.86 [-2.28, -1.44]
Tian 2014	18.21	7.82	30	17.13	6.43	28	4.8%	0.15 [-0.37, 0.66]
Tian 2021	14.37	4.6	30	25.45	7.24	30	4.7%	-1.80 [-2.41, -1.20]
Wanga 2019	21.31	3.28	75	25.14	4.37	75	4.9%	-0.99 [-1.33, -0.65]
Wu 2012	24.72	7.41	42	15.77	3.81	21	4.7%	1.37 [0.79, 1.95]
Ye 2012	21.63	3.19	34	22.57	3.91	34	4.8%	-0.26 [-0.74, 0.22]
Zeng2023	22.43	1.2	54	26.38	1.31	54	4.7%	-3.12 [-3.69, -2.55]
Zhang2022	15.54	2.25	44	19.69	2.63	42	4.8%	-1.68 [-2.18, -1.19]
Zhanga 2020	12.18	1.96	43	15.01	2.46	43	4.8%	-1.26 [-1.73, -0.80]
Zhao2022	11.87	2.35	40	15.21	2.81	40	4.8%	-1.28 [-1.76, -0.79]
**Subtotal (95% CI)**			**697**			**672**	**71.9%**	**-1.51 [-2.11, -0.91]**
Heterogeneity: Tau² = 1.33; Chi² = 305.69, df = 14 (P < 0.00001); I² = 95%								
Test for overall effect: Z = 4.96 (P < 0.00001)								
**4.5.2 >12weeks**								
Li 2021	21.5	3.74	25	38.74	4.71	25	4.2%	-3.99 [-4.98, -3.00]
Liu 2014	26.3	5.2	28	27.4	5.1	28	4.8%	-0.21 [-0.74, 0.31]
Wang 2018	26.7	6.7	40	27.6	4.3	40	4.9%	-0.16 [-0.60, 0.28]
Wang2023	0	0	0	0	0	0		Not estimable
Wangb 2019	16.1	3.8	28	28.9	7.3	28	4.6%	-2.17 [-2.84, -1.50]
Xie 2019	13.7	2.3	50	17.6	2.5	50	4.8%	-1.61 [-2.06, -1.16]
Ying 2018	24.21	0.87	47	26.65	1.04	47	4.8%	-2.52 [-3.07, -1.98]
**Subtotal (95% CI)**			**218**			**218**	**28.1%**	**-1.73 [-2.74, -0.72]**
Heterogeneity: Tau² = 1.50; Chi² = 98.60, df = 5 (P < 0.00001); I² = 95%								
Test for overall effect: Z = 3.36 (P = 0.0008)								
**Total (95% CI)**			**915**			**890**	**100.0%**	**-1.57 [-2.07, -1.07]**
Heterogeneity: Tau² = 1.29; Chi² = 404.94, df = 20 (P < 0.00001); I² = 95%								
Test for overall effect: Z = 6.16 (P < 0.00001)								
Test for subgroup differences: Chi² = 0.13, df = 1 (P = 0.71), I² = 0%								

Std. Mean Difference IV, Random, 95% CI

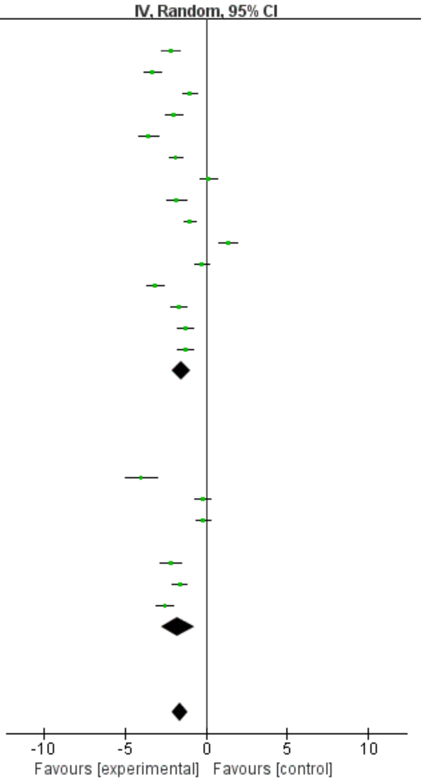

Fig.S4E

Study or Subgroup	Experimental Mean	Experimental SD	Experimental Total	Control Mean	Control SD	Control Total	Weight	Std. Mean Difference IV, Random, 95% CI
**5.1.1 2-3 capsule**								
Cui 2020	8.92	2.98	66	4.12	1.01	34	6.8%	1.91 [1.41, 2.40]
Huang 2018	3.56	0.3	50	6.62	0.29	50	6.0%	-10.29 [-11.80, -8.78]
Lin2024	0	0	0	0	0	0		Not estimable
Sun 2011	2.4	1.4	69	9.3	3.5	33	6.7%	-2.99 [-3.58, -2.40]
Sun 2012	3.1	1.5	53	9.8	2.5	49	6.7%	-3.26 [-3.86, -2.66]
Zhu 2019	6.42	1.34	45	9.92	1.63	45	6.8%	-2.33 [-2.87, -1.79]
**Subtotal (95% CI)**			**283**			**211**	**33.0%**	**-3.32 [-6.03, -0.60]**
Heterogeneity: Tau² = 9.43; Chi² = 379.08, df = 4 (P < 0.00001); I² = 99%								
Test for overall effect: Z = 2.39 (P = 0.02)								
**5.1.2 4-6 capsule**								
Chen 2017	3.15	1.06	25	4.85	1.62	25	6.7%	-1.22 [-1.83, -0.61]
Gao 2019	4.56	0.87	41	6.73	1.08	41	6.7%	-2.19 [-2.74, -1.64]
Li2023	0	0	0	0	0	0		Not estimable
Tang 2024	0	0	0	0	0	0		Not estimable
Wang2023	6.72	1.48	30	7.39	1.46	30	6.8%	-0.45 [-0.96, 0.06]
Wangb 2019	5.1	1.2	28	9.7	1.4	28	6.6%	-3.48 [-4.33, -2.63]
Wu 2012	7.5	1.99	42	5.01	1.37	21	6.7%	1.36 [0.78, 1.94]
Zeng2023	0	0	0	0	0	0		Not estimable
Zhang2022	6.47	1.53	44	8.12	2.21	42	6.8%	-0.86 [-1.31, -0.42]
Zhanga 2020	6.3	0.83	43	9.18	1.21	43	6.7%	-2.75 [-3.35, -2.15]
Zhao2022	1.28	0.57	40	2.79	0.88	40	6.8%	-2.02 [-2.56, -1.47]
Zhu 2020	2.06	0.34	39	5.19	0.71	39	6.5%	-5.57 [-6.57, -4.57]
**Subtotal (95% CI)**			**332**			**309**	**60.3%**	**-1.87 [-2.93, -0.82]**
Heterogeneity: Tau² = 2.49; Chi² = 228.75, df = 8 (P < 0.00001); I² = 97%								
Test for overall effect: Z = 3.49 (P = 0.0005)								
**5.1.3 >6 capsule**								
Liu 2014	6.2	2.3	28	6.8	2.1	28	6.8%	-0.27 [-0.80, 0.26]
**Subtotal (95% CI)**			**28**			**28**	**6.8%**	**-0.27 [-0.80, 0.26]**
Heterogeneity: Not applicable								
Test for overall effect: Z = 1.00 (P = 0.32)								
**Total (95% CI)**			**643**			**548**	**100.0%**	**-2.22 [-3.24, -1.20]**
Heterogeneity: Tau² = 3.97; Chi² = 628.64, df = 14 (P < 0.00001); I² = 98%								
Test for overall effect: Z = 4.25 (P < 0.0001)								
Test for subgroup differences: Chi² = 10.90, df = 2 (P = 0.004), I² = 81.6%								

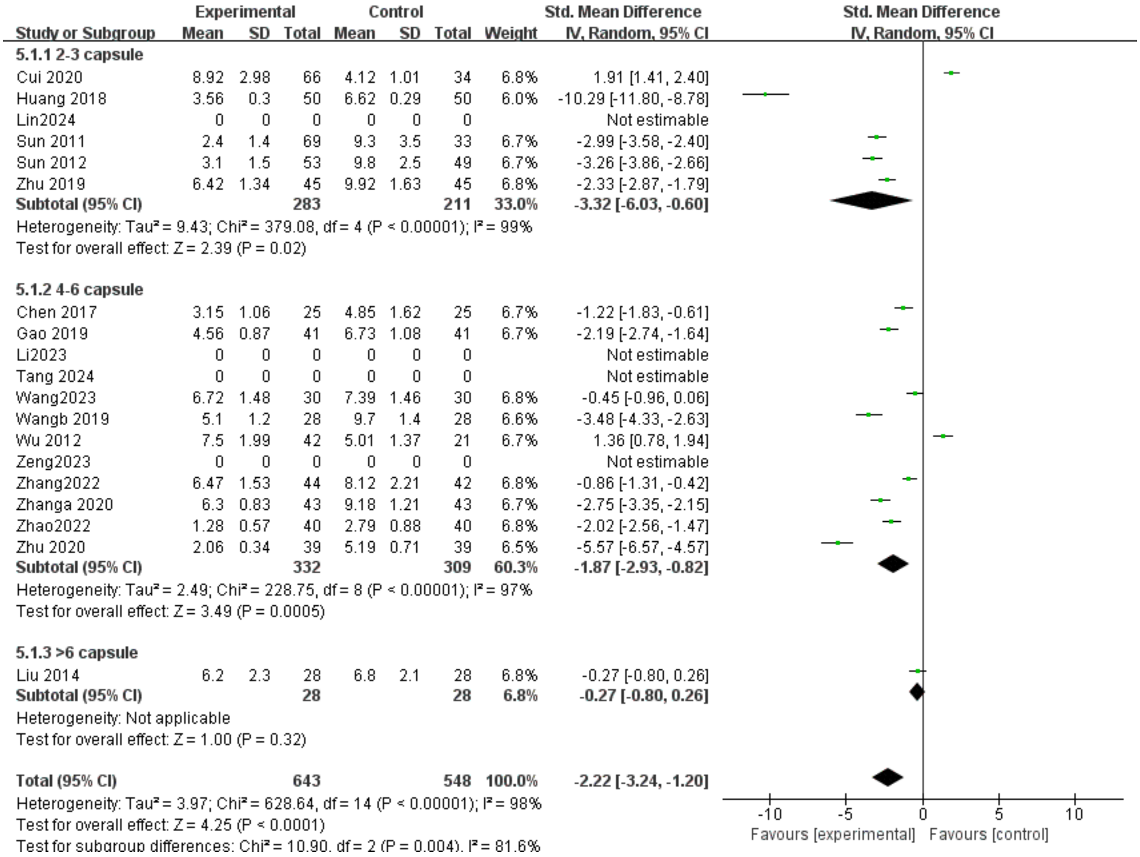

Fig.S5A

Study or Subgroup	Experimental Mean	Experimental SD	Experimental Total	Control Mean	Control SD	Control Total	Weight	Std. Mean Difference IV, Random, 95% CI
**5.2.1 2-3 capsule**								
Cui 2020	35.42	4.68	66	42.41	0	34		Not estimable
Huang 2018	39.65	6.56	50	35.62	6.23	50	5.4%	0.63 [0.22, 1.03]
Lin2024	0	0	0	0	0	0		Not estimable
Sun 2011	36.9	10.8	69	29.8	7.5	33	5.4%	0.71 [0.29, 1.14]
Sun 2012	36	10	53	30	8	49	5.4%	0.65 [0.26, 1.05]
Tian 2014	29.51	4.97	30	30.16	5.81	28	5.2%	-0.12 [-0.63, 0.40]
Tian 2021	35.31	4.41	30	33.11	5.27	30	5.2%	0.45 [-0.07, 0.96]
Yang 2019	39.12	5.98	36	34.59	6.02	36	5.3%	0.75 [0.27, 1.23]
Ying 2018	42.31	1.78	47	39.46	1.51	47	5.3%	1.71 [1.24, 2.19]
Zhu 2019	42.19	4.83	45	39.73	4.91	45	5.4%	0.50 [0.08, 0.92]
**Subtotal (95% CI)**			**426**			**352**	**42.4%**	**0.66 [0.34, 0.99]**
Heterogeneity: Tau² = 0.16; Chi² = 29.02, df = 7 (P = 0.0001); I² = 76%								
Test for overall effect: Z = 4.02 (P < 0.0001)								
**5.2.2 4-6 capsule**								
Gao 2019	43.25	1.45	41	40.76	1.8	41	5.2%	1.51 [1.02, 2.00]
Li2023	44.82	7.28	65	41.19	5.31	65	5.5%	0.57 [0.22, 0.92]
Tang 2024	0	0	0	0	0	0		Not estimable
Wang2023	0	0	0	0	0	0		Not estimable
Wu 2012	32.56	11.38	42	33.12	9.66	21	5.2%	-0.05 [-0.57, 0.47]
Zeng2023	36.29	1.25	54	31.14	1.42	54	4.9%	3.82 [3.18, 4.46]
Zhang2022	41.19	3.85	44	35.49	4.67	42	5.3%	1.32 [0.85, 1.79]
Zhangb 2020	38.43	3.08	47	31.61	2.17	47	5.1%	2.54 [1.99, 3.09]
Zhao2022	41.17	5.46	40	40.03	5.21	40	5.3%	0.21 [-0.23, 0.65]
Zhu 2020	45.52	4.67	39	41.68	4.73	39	5.3%	0.81 [0.35, 1.27]
**Subtotal (95% CI)**			**372**			**349**	**41.7%**	**1.33 [0.56, 2.09]**
Heterogeneity: Tau² = 1.15; Chi² = 143.68, df = 7 (P < 0.00001); I² = 95%								
Test for overall effect: Z = 3.40 (P = 0.0007)								
**5.2.3 >6 capsule**								
Liu 2014	41.9	2.7	28	41.2	2.3	28	5.1%	0.28 [-0.25, 0.80]
Wang 2016	42.1	3.2	30	41.5	3.4	30	5.2%	0.18 [-0.33, 0.69]
Wanga 2019	42.36	2.68	75	38.46	3.27	75	5.5%	1.30 [0.94, 1.65]
**Subtotal (95% CI)**			**133**			**133**	**15.8%**	**0.60 [-0.17, 1.38]**
Heterogeneity: Tau² = 0.41; Chi² = 17.19, df = 2 (P = 0.0002); I² = 88%								
Test for overall effect: Z = 1.52 (P = 0.13)								
**Total (95% CI)**			**931**			**834**	**100.0%**	**0.92 [0.57, 1.28]**
Heterogeneity: Tau² = 0.56; Chi² = 202.99, df = 18 (P < 0.00001); I² = 91%								
Test for overall effect: Z = 5.12 (P < 0.00001)								
Test for subgroup differences: Chi² = 2.59, df = 2 (P = 0.27), I² = 22.7%								

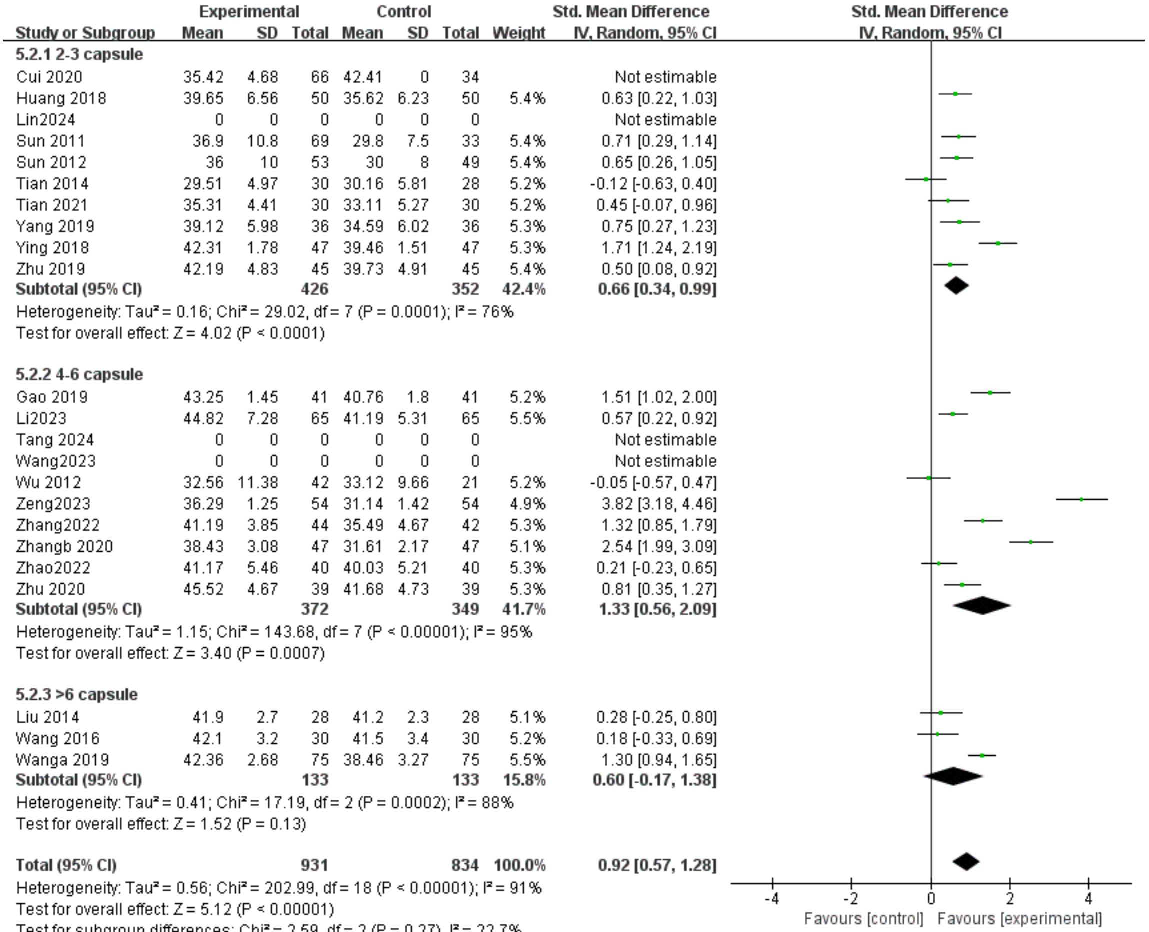

Fig.S5B

	Experimental			Control				Std. Mean Difference
**Study or Subgroup**	**Mean**	**SD**	**Total**	**Mean**	**SD**	**Total**	**Weight**	**IV, Random, 95% CI**
**5.3.1 2-3 capsule**								
He 2021	106.5	9.44	38	95.69	8.31	38	5.3%	1.20 [0.71, 1.69]
Lin2024	115.53	12.53	40	108.64	11.44	40	5.4%	0.57 [0.12, 1.02]
Ling 2021	102.35	7.83	41	92.67	7.65	41	5.3%	1.24 [0.76, 1.71]
Ren 2021	147.91	6.45	55	125.57	6.41	55	5.0%	3.45 [2.85, 4.05]
Sun 2011	123.1	19	69	97.4	9.2	33	5.3%	1.54 [1.08, 2.01]
Sun 2012	119	12	53	96	9	49	5.3%	2.14 [1.65, 2.63]
Tian 2014	89.4	11.61	30	88.16	12.64	28	5.2%	0.10 [-0.41, 0.62]
Tian 2021	89.2	11.56	30	77.18	12.24	30	5.2%	1.00 [0.46, 1.54]
Yang 2019	93.74	11.28	36	84.01	9.83	36	5.3%	0.91 [0.42, 1.40]
Ying 2018	98.42	3.25	47	94.25	2.72	47	5.3%	1.38 [0.93, 1.83]
Zhu 2019	103.64	11.09	45	94.86	10.32	45	5.4%	0.81 [0.38, 1.24]
**Subtotal (95% CI)**			**484**			**442**	**57.9%**	**1.29 [0.83, 1.76]**
Heterogeneity: Tau² = 0.56; Chi² = 101.52, df = 10 (P < 0.00001); I² = 90%								
Test for overall effect: Z = 5.44 (P < 0.00001)								
**5.3.2 4-6 capsule**								
Gao 2019	108.42	9.36	41	94.65	4.78	41	5.2%	1.84 [1.32, 2.36]
Li2023	92.83	8.24	65	86.1	9.75	65	5.5%	0.74 [0.39, 1.10]
Tang 2024	0	0	0	0	0	0		Not estimable
Wang2023	89.07	9.26	30	83.36	12.3	30	5.2%	0.52 [0.00, 1.03]
Ye 2012	104.69	12.46	34	106.72	13.09	34	5.3%	-0.16 [-0.63, 0.32]
Zeng2023	109.38	6.24	54	95.21	6.28	54	5.3%	2.25 [1.76, 2.73]
Zhang2022	0	0	0	0	0	0		Not estimable
Zhao2022	0	0	0	0	0	0		Not estimable
Zhu 2020	108.67	8.51	39	97.85	8.13	39	5.3%	1.29 [0.80, 1.78]
**Subtotal (95% CI)**			**263**			**263**	**31.8%**	**1.08 [0.39, 1.76]**
Heterogeneity: Tau² = 0.67; Chi² = 64.66, df = 5 (P < 0.00001); I² = 92%								
Test for overall effect: Z = 3.08 (P = 0.002)								
**5.3.3 >6 capsule**								
Liu 2014	102.4	8.3	28	98.7	9.4	28	5.2%	0.41 [-0.12, 0.94]
Wang 2016	102.6	8.5	30	99.8	9.6	30	5.2%	0.30 [-0.20, 0.81]
**Subtotal (95% CI)**			**58**			**58**	**10.4%**	**0.36 [-0.01, 0.72]**
Heterogeneity: Tau² = 0.00; Chi² = 0.08, df = 1 (P = 0.78); I² = 0%								
Test for overall effect: Z = 1.90 (P = 0.06)								
**Total (95% CI)**			**805**			**763**	**100.0%**	**1.13 [0.77, 1.48]**
Heterogeneity: Tau² = 0.56; Chi² = 185.82, df = 18 (P < 0.00001); I² = 90%								
Test for overall effect: Z = 6.23 (P < 0.00001)								
Test for subgroup differences: Chi² = 10.51, df = 2 (P = 0.005), I² = 81.0%								

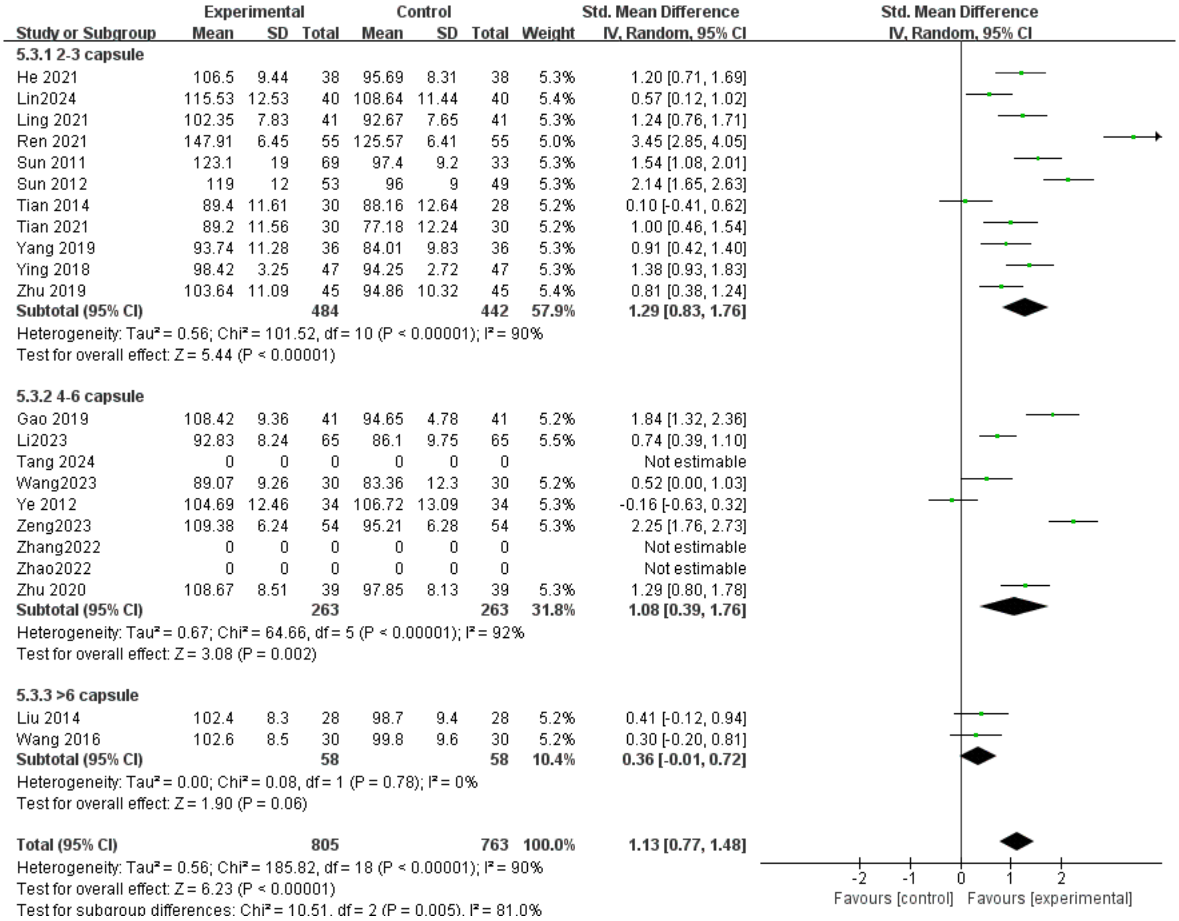

Fig.S5C

Study or Subgroup	Experimental Mean	Experimental SD	Experimental Total	Control Mean	Control SD	Control Total	Weight	Std. Mean Difference IV, Random, 95% CI
**5.4.1 2-3 capsule**								
Lin2024	315.44	25.66	40	362.18	21.35	40	4.6%	-1.96 [-2.50, -1.42]
Ling 2021	248.25	22.34	41	301.64	30.47	41	4.6%	-1.98 [-2.51, -1.45]
Ren 2021	287.89	21.14	55	325.41	21.12	55	4.7%	-1.76 [-2.21, -1.32]
Tian 2014	945.76	311.54	30	988.94	321.58	28	4.6%	-0.13 [-0.65, 0.38]
Tian 2021	341.4	108.41	30	660.71	185.27	30	4.5%	-2.08 [-2.71, -1.44]
Ying 2018	845.29	7.56	47	885.13	8.98	47	4.2%	-4.76 [-5.56, -3.96]
**Subtotal (95% CI)**			**243**			**241**	**27.1%**	**-2.08 [-3.07, -1.10]**
Heterogeneity: Tau² = 1.43; Chi² = 93.66, df = 5 (P < 0.00001); I² = 95%								
Test for overall effect: Z = 4.15 (P < 0.0001)								
**5.4.2 4-6 capsule**								
Gao 2019	175.52	16.85	41	234.51	23.27	41	4.5%	-2.88 [-3.50, -2.25]
Li 2021	604.3	22.42	25	697.88	22.25	25	3.9%	-4.12 [-5.13, -3.12]
Li2023	324.75	84.6	65	385.4	93.47	65	4.7%	-0.68 [-1.03, -0.32]
Tang 2024	385.73	102.62	63	423.64	103.55	63	4.7%	-0.37 [-0.72, -0.01]
Wang 2018	765	68	40	762	54	40	4.7%	0.05 [-0.39, 0.49]
Wang2023	0	0	0	0	0	0		Not estimable
Wangb 2019	697.5	53.1	28	849.7	60.3	28	4.3%	-2.64 [-3.37, -1.91]
Wu 2012	783.9	77.06	42	694.54	52.33	21	4.5%	1.26 [0.69, 1.83]
Yao 2016	546.58	109.09	17	541.67	114.78	18	4.4%	0.04 [-0.62, 0.71]
Ye 2012	575.69	29.97	34	581.27	30.21	34	4.6%	-0.18 [-0.66, 0.29]
Zeng2023	831.24	61.39	54	892.53	55.1	54	4.7%	-1.04 [-1.45, -0.64]
Zhang2022	157.95	12.47	44	188.76	19.68	42	4.6%	-1.86 [-2.37, -1.35]
Zhanga 2020	453.77	48.76	43	510.33	55.45	43	4.6%	-1.07 [-1.53, -0.62]
Zhao2022	451.46	51.24	40	497.65	59.38	40	4.6%	-0.82 [-1.28, -0.37]
**Subtotal (95% CI)**			**536**			**514**	**59.0%**	**-1.06 [-1.63, -0.48]**
Heterogeneity: Tau² = 1.02; Chi² = 211.50, df = 12 (P < 0.00001); I² = 94%								
Test for overall effect: Z = 3.62 (P = 0.0003)								
**5.4.3 >6 capsule**								
Liu 2014	916.8	102.7	28	923.3	107.4	28	4.6%	-0.06 [-0.58, 0.46]
Wanga 2019	832.52	84.25	75	902.35	96.57	75	4.7%	-0.77 [-1.10, -0.43]
Xie 2019	314.5	18.5	50	357.4	20.1	50	4.6%	-2.20 [-2.70, -1.70]
**Subtotal (95% CI)**			**153**			**153**	**13.9%**	**-1.01 [-2.11, 0.09]**
Heterogeneity: Tau² = 0.89; Chi² = 36.31, df = 2 (P < 0.00001); I² = 94%								
Test for overall effect: Z = 1.80 (P = 0.07)								
**Total (95% CI)**			**932**			**908**	**100.0%**	**-1.33 [-1.79, -0.87]**
Heterogeneity: Tau² = 1.13; Chi² = 399.90, df = 21 (P < 0.00001); I² = 95%								
Test for overall effect: Z = 5.66 (P < 0.00001)								
Test for subgroup differences: Chi² = 3.38, df = 2 (P = 0.18), I² = 40.9%								

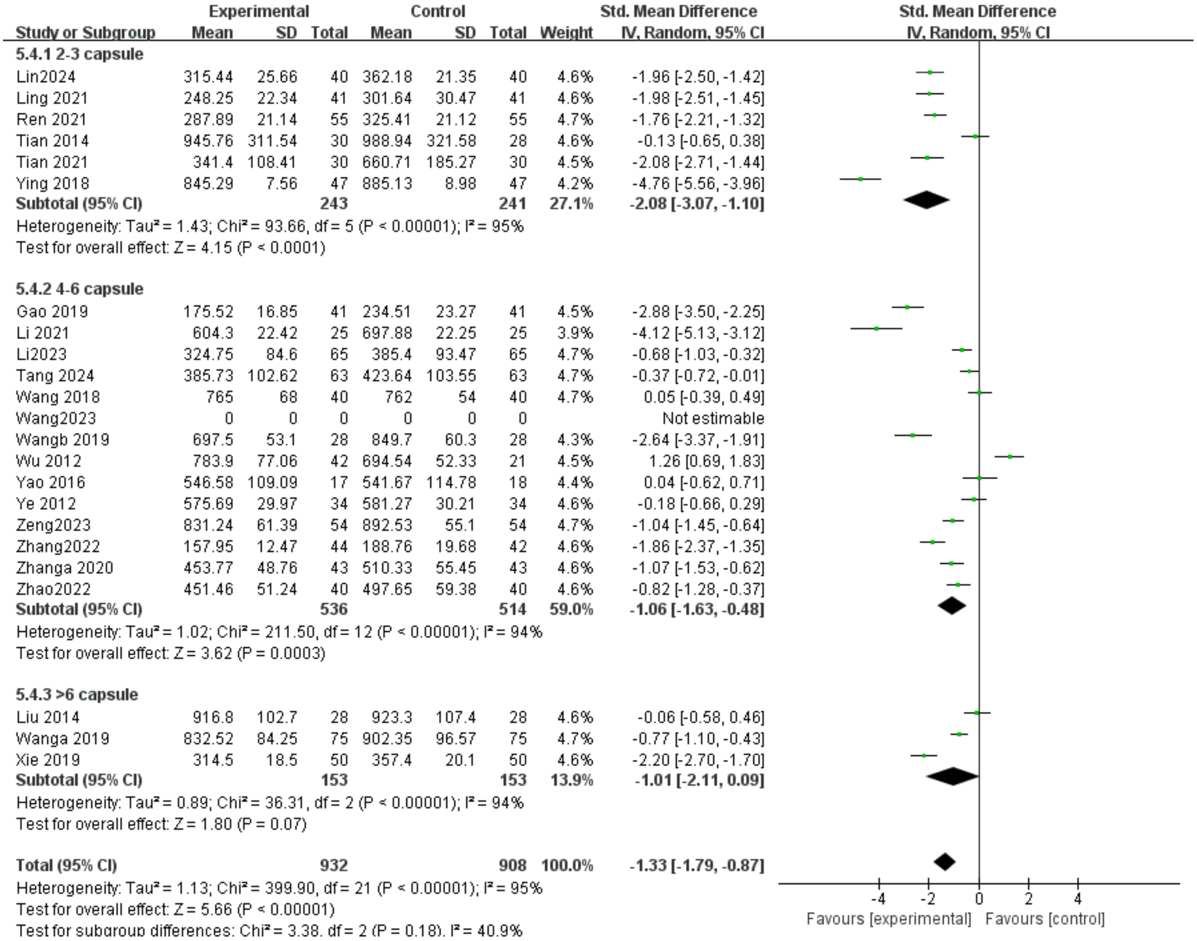

Fig.S5D

Study or Subgroup	Experimental Mean	Experimental SD	Experimental Total	Control Mean	Control SD	Control Total	Weight	Std. Mean Difference IV, Random, 95% CI
**5.5.1 2-3 capsule**								
Lin2024	14.27	2.37	40	16.15	1.18	40	4.8%	-0.99 [-1.46, -0.53]
Ling 2021	11.33	2.05	41	15.78	2.34	41	4.8%	-2.00 [-2.54, -1.47]
Ren 2021	12.84	1.69	55	18.71	1.62	55	4.7%	-3.52 [-4.12, -2.92]
Tian 2014	18.21	7.82	30	17.13	6.43	28	4.8%	0.15 [-0.37, 0.66]
Tian 2021	14.37	4.6	30	25.45	7.24	30	4.7%	-1.80 [-2.41, -1.20]
Ying 2018	24.21	0.87	47	26.65	1.04	47	4.8%	-2.52 [-3.07, -1.98]
**Subtotal (95% CI)**			**243**			**241**	**28.6%**	**-1.78 [-2.78, -0.77]**
Heterogeneity: Tau² = 1.49; Chi² = 102.97, df = 5 (P < 0.00001); I² = 95%								
Test for overall effect: Z = 3.47 (P = 0.0005)								
**5.5.2 4-6 capsule**								
Gao 2019	13.26	2.87	41	18.98	2.33	41	4.8%	-2.17 [-2.72, -1.62]
Li 2021	21.5	3.74	25	38.74	4.71	25	4.2%	-3.99 [-4.98, -3.00]
Li2023	12.15	2.96	65	19.75	1.26	65	4.8%	-3.32 [-3.86, -2.79]
Tang 2024	13.82	2.19	63	17.94	2.21	63	4.9%	-1.86 [-2.28, -1.44]
Wang 2018	26.7	6.7	40	27.6	4.3	40	4.9%	-0.16 [-0.60, 0.28]
Wang2023	0	0	0	0	0	0		Not estimable
Wangb 2019	16.1	3.8	28	28.9	7.3	28	4.6%	-2.17 [-2.84, -1.50]
Wu 2012	24.72	7.41	42	15.77	3.81	21	4.7%	1.37 [0.79, 1.95]
Ye 2012	21.63	3.19	34	22.57	3.91	34	4.8%	-0.26 [-0.74, 0.22]
Zeng2023	22.43	1.2	54	26.38	1.31	54	4.7%	-3.12 [-3.69, -2.55]
Zhang2022	15.54	2.25	44	19.69	2.63	42	4.8%	-1.68 [-2.18, -1.19]
Zhanga 2020	12.18	1.96	43	15.01	2.46	43	4.8%	-1.26 [-1.73, -0.80]
Zhao2022	11.87	2.35	40	15.21	2.81	40	4.8%	-1.28 [-1.76, -0.79]
**Subtotal (95% CI)**			**519**			**496**	**56.9%**	**-1.64 [-2.39, -0.89]**
Heterogeneity: Tau² = 1.68; Chi² = 270.62, df = 11 (P < 0.00001); I² = 96%								
Test for overall effect: Z = 4.27 (P < 0.0001)								
**5.5.3 >6 capsule**								
Liu 2014	26.3	5.2	28	27.4	5.1	28	4.8%	-0.21 [-0.74, 0.31]
Wanga 2019	21.31	3.28	75	25.14	4.37	75	4.9%	-0.99 [-1.33, -0.65]
Xie 2019	13.7	2.3	50	17.6	2.5	50	4.8%	-1.61 [-2.06, -1.16]
**Subtotal (95% CI)**			**153**			**153**	**14.6%**	**-0.95 [-1.65, -0.25]**
Heterogeneity: Tau² = 0.33; Chi² = 15.66, df = 2 (P = 0.0004); I² = 87%								
Test for overall effect: Z = 2.64 (P = 0.008)								
**Total (95% CI)**			**915**			**890**	**100.0%**	**-1.57 [-2.07, -1.07]**
Heterogeneity: Tau² = 1.29; Chi² = 404.94, df = 20 (P < 0.00001); I² = 95%								
Test for overall effect: Z = 6.16 (P < 0.00001)								
Test for subgroup differences: Chi² = 2.51, df = 2 (P = 0.29), I² = 20.2%								

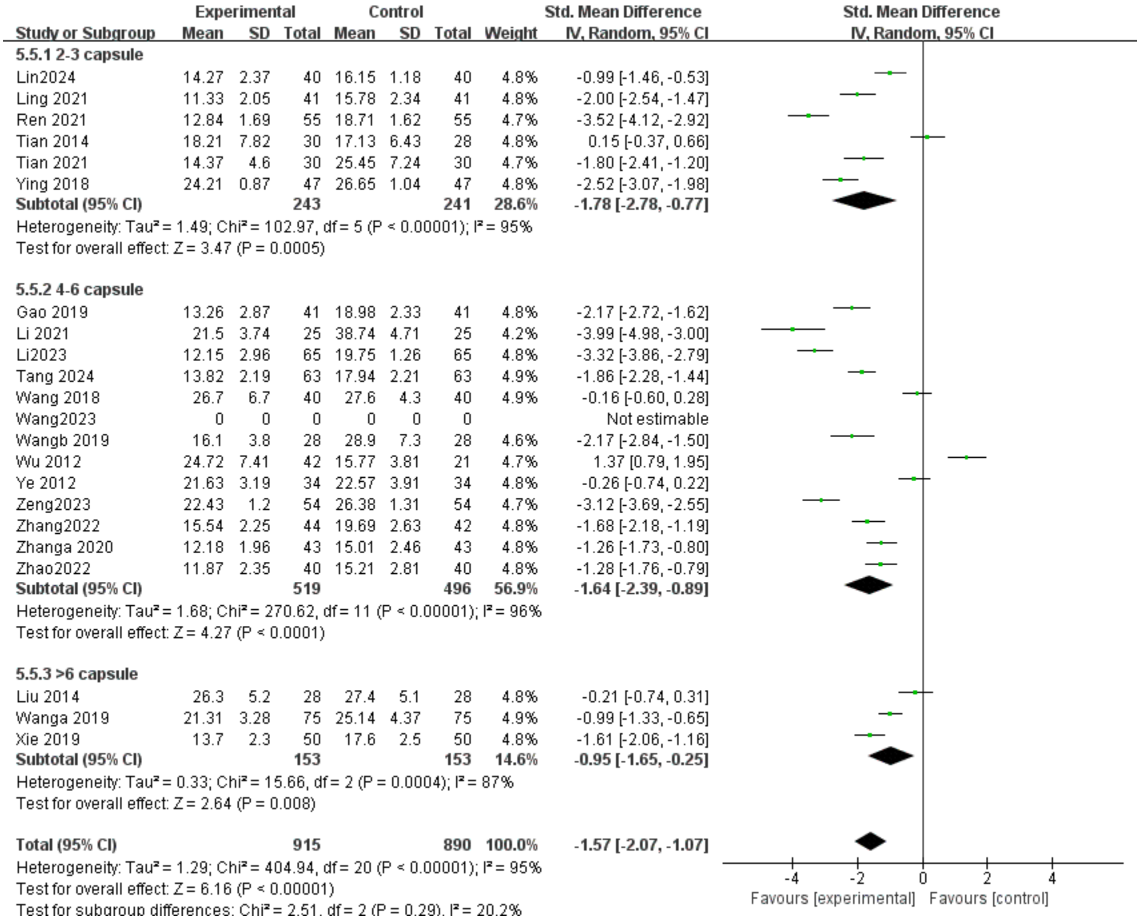

Fig.S5E
